# Immunologic and inflammatory consequences of SARS-CoV-2 infection and its implications in renal disease

**DOI:** 10.3389/fimmu.2024.1376654

**Published:** 2025-02-12

**Authors:** Hiam Naiditch, Michael R. Betts, H. Benjamin Larman, Moshe Levi, Avi Z. Rosenberg

**Affiliations:** ^1^ Department of Pulmonary, Allergy, Critical Care and Sleep Medicine, University of Pittsburgh, Pittsburgh, PA, United States; ^2^ Department of Microbiology and Institute of Immunology, Perelman School of Medicine, University of Pennsylvania, Philadelphia, PA, United States; ^3^ Institute for Cell Engineering, Division of Immunology, Department of Pathology, Johns Hopkins University, Baltimore, MD, United States; ^4^ Department of Biochemistry and Molecular & Cellular Biology, Georgetown University, Washington, DC, United States; ^5^ Department of Pathology, Johns Hopkins University, Baltimore, MD, United States

**Keywords:** SARS-CoV-2, COVID-19, PASC, long COVID, inflammasome, inflammation, AKI

## Abstract

The emergence of the COVID-19 pandemic made it critical to understand the immune and inflammatory responses to the SARS-CoV-2 virus. It became increasingly recognized that the immune response was a key mediator of illness severity and that its mechanisms needed to be better understood. Early infection of both tissue and immune cells, such as macrophages, leading to pyroptosis-mediated inflammasome production in an organ system critical for systemic oxygenation likely plays a central role in the morbidity wrought by SARS-CoV-2. Delayed transcription of Type I and Type III interferons by SARS-CoV-2 may lead to early disinhibition of viral replication. Cytokines such as interleukin-1 (IL-1), IL-6, IL-12, and tumor necrosis factor α (TNFα), some of which may be produced through mechanisms involving nuclear factor kappa B (NF-κB), likely contribute to the hyperinflammatory state in patients with severe COVID-19. Lymphopenia, more apparent among natural killer (NK) cells, CD8+ T-cells, and B-cells, can contribute to disease severity and may reflect direct cytopathic effects of SARS-CoV-2 or end-organ sequestration. Direct infection and immune activation of endothelial cells by SARS-CoV-2 may be a critical mechanism through which end-organ systems are impacted. In this context, endovascular neutrophil extracellular trap (NET) formation and microthrombi development can be seen in the lungs and other critical organs throughout the body, such as the heart, gut, and brain. The kidney may be among the most impacted extrapulmonary organ by SARS-CoV-2 infection owing to a high concentration of ACE2 and exposure to systemic SARS-CoV-2. In the kidney, acute tubular injury, early myofibroblast activation, and collapsing glomerulopathy in select populations likely account for COVID-19-related AKI and CKD development. The development of COVID-19-associated nephropathy (COVAN), in particular, may be mediated through IL-6 and signal transducer and activator of transcription 3 (STAT3) signaling, suggesting a direct connection between the COVID-19-related immune response and the development of chronic disease. Chronic manifestations of COVID-19 also include systemic conditions like Multisystem Inflammatory Syndrome in Children (MIS-C) and Adults (MIS-A) and post-acute sequelae of COVID-19 (PASC), which may reflect a spectrum of clinical presentations of persistent immune dysregulation. The lessons learned and those undergoing continued study likely have broad implications for understanding viral infections’ immunologic and inflammatory consequences beyond coronaviruses.

## 1 Introduction

On March 11, 2020, as healthcare systems worldwide saw rising SARS-CoV-2 cases, COVID-19 was declared a pandemic (1). The infectivity of the virus and the severity of COVID-19 in many patients led to the accelerated study of the disease (2). It became increasingly apparent that the immune milieu generated by SARS-CoV-2 was unique, even among similar coronaviruses (3). Peripheral lymphopenia, elevated inflammatory markers (4), endothelial damage, and microthrombosis characterized some early findings in COVID-19 (5). In the years that followed, the immune response to COVID-19 has been further elucidated, allowing for the identification of effective vaccines (6, 7), monoclonal antibodies (8), and other therapies (9, 10). The emergence of syndromes associated with post-infectious immune dysregulation such as Multisystem Inflammatory Syndrome in children (MIS-C) (11, 12) or adults (MIS-A) (13, 14) further propelled the study of the disease. A compelling association with autoimmune disease, perhaps related to the development of autoantibodies in the presence of SARS-CoV-2-mediated pyroptosis, further exemplified the complex interplay between the immunologic response in COVID-19 and chronic disease (15).

Among the organ systems studied, a high concentration of ACE2 in a highly vascular structure readily exposed to systemic pathogens highlights the human kidney as a unique model for the systemic effects of SARS-CoV-2 (16). Prior to infection, there is a paucity of immune cells in the human kidney; most of these are CD4+ and CD8+ T cells, with a smaller percentage of NK cells, B cells (17), and CD14+, CD16+ and CD68+ myeloid cells (18). Following infection with SARS-CoV-2, an upregulation in proinflammatory genes such as HSPA1A in podocytes and JUN1 in mesenchymal clusters (19) can accompany selective immune suppression of lymphocytes mediated through T-cell immunoglobulin and mucin-domain containing-3 (TIM-3) and Programmed cell death protein 1 (PD-1) (20). Resident immune cells may mediate inflammation by TNFα release, IL-34-mediated necrosis, and NLRP3 inflammasome production (266). Myeloid cell activation is a hallmark of COVID-19 and is associated with immune dysregulation in COVID-19, particularly in severe disease (21). Moreover, early post-mortem studies revealed compelling evidence for direct infection of predominantly ACE2-positive renal tubular cells by SARS-CoV-2 (16). Intriguingly, the upregulation of TGF-β, PI3K/Akt, MAPK, and WNT signaling can be associated with tubule interstitial fibrosis and may also point to a mechanism of COVID-19-related CKD (19).

The following represents a comprehensive review of the immunologic and inflammatory consequences of SARS-CoV-2 infection, synthesizing the molecular immune response to the acute and chronic end-organ dysfunction in both acute and chronic forms of COVID-19. Among the organ systems studied, particular attention is paid to the kidney and recently described pathophysiology in COVID-19.

### 1.1 Initial infection with SARS-CoV-2

SARS-CoV-2 infection occurs predominantly in the upper respiratory tract via ACE2 and TMPRSS2.

Coronaviruses likely originated in bats and rodents and eventually evolved to affect other animals and humans ￼. Their pathogenicity was not fully appreciated until 2002, when the sudden acute respiratory syndrome (SARS) broke out in Guangdong Province, China (22). Since that time, coronaviruses have been implicated in Middle East respiratory syndrome (MERS) and other less overtly severe clinical entities (22). Approximately seven years after MERS, SARS-CoV-2 emerged as a public health threat, spurring research into its structure, infectivity, and the immune response it provokes.

SARS-CoV-2 is a betacoronavirus with an envelope containing an envelope (E) protein, a membrane (M) protein, and a spike (S) protein interspersed within a lipid membrane (5). The nucleus contains a positive-sense single-stranded RNA molecule bound to the nucleocapsid (N) protein (23). The bulk of the ssRNA has two open reading frames at the 5’ end, ORF1a and ORF1b, which are the transcriptional precursors of the viral replication and transcription complex (RTC) (23). SARS-CoV-2 is transmitted by respiratory and aerosolized droplets from infected individuals actively shedding the virus (24–26). Virions can contact areas of high angiotensin-converting enzyme 2 (ACE2) and transmembrane protease serine 2 (TMPRSS2) co-expression, namely in goblet secretory cells in the nasopharynx, epithelial cells of the oral mucosa, airway, and alveoli including Type II pneumocytes, and lung macrophages (27, 28). Viral entry into host cells is facilitated by binding the S protein to ACE2, followed by proteolytic cleavage at the S1/S2 and S’ sites by TMPRSS2, a process that allows for virus-host membrane fusion (29). Expression of ACE2 and TMPRSS2 has also been noted in endothelial cells, enterocytes (27), and podocytes in the kidney glomerulus (5).

**Figure 1:**
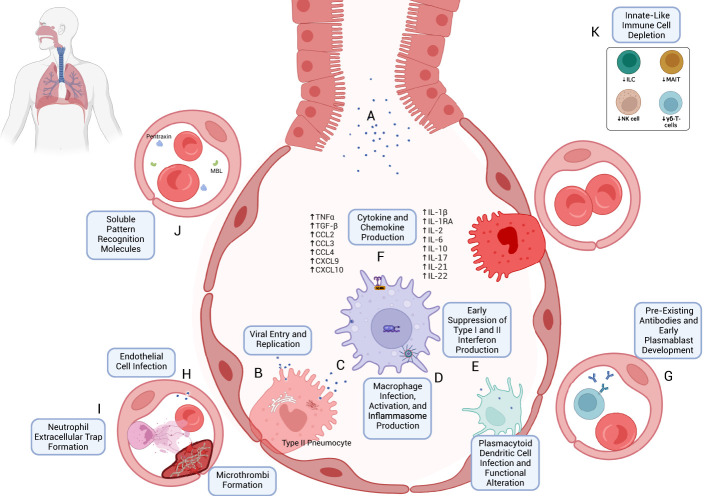
Early immune response to SARS-CoV-2 infection. **(A)** SARS-CoV-2 virions are inhaled through respiratory and aerosolized droplets (24–26). Infection of susceptible cell types, particularly those bearing ACE2 and TMPRSS, such as Type II pneumocytes, can occur (27, 28). **(B)** Viral entry can occur through mechanisms including host-membrane fusion (29). Entry is followed by the release and translation of ORF1a and ORF1b of SARS-CoV-2 ssRNA, formation of the viral replication and transcription complex (23), and production of new SARS-CoV-2 virions, which are eventually exocytosed. **(C)** Resident macrophages are likely among the first immune cells to encounter SARS-CoV-2 through both direct infection and indirect immune activation. As with other cells, TLR- and RLR-mediated recognition are accompanied by downstream effects through Myd88 activation, TRIF binding (43, 45, 50), MAVS activation (45), and NF-κB activation (45, 52). **(D)** Production of Type I and Type III interferons appears to be blunted in the early immune response (42, 89) despite their antiviral potential. **(E)** Infection and activation with SARS-CoV-2 may induce inflammasome production and macrophage pyroptosis, which may be a key early driver of a heightened immune response following SARS-CoV-2 infection (57). **(F)** One such mechanism is through cytokine and chemokine release (70, 103, 104), including TNFα, TGF-β, CCL2/3, CXCL9/10, IL-1β, IL-2, IL-6, IL-10, IL-17, IL-21, and IL-22 (see text for references). **(G)** The early immune response may also be mediated by pre-existing cross-reactive antibodies (174–177) and plasmablast development (173). **(H)** Endothelial cell infection may also occur (207); endothelial cell activation may be associated with a hypercoagulable state (210, 211). **(I)** Early neutrophil responses include the production of immature neutrophils through emergency myelopoiesis (115, 400) and neutrophil extracellular trap (NET) formation, which may also contribute to microvascular thrombosis (111, 117). **(J)** Soluble pattern recognition molecules such as mannose-binding lectin (MBL) and pentraxin three can bind to SARS-CoV-2 spike and nucleocapsid proteins, respectively (96). **(K)** Lymphopenia, which often accompanies COVID-19 (408), can include the peripheral depletion of cell types such as Innate Lymphoid Cells (ILC) (126, 127), MAIT (127), NK-cells (124, 398), and γδ-T-cells (136).

The importance of ACE2 has been confirmed through GWAS identification of variants (such as X-linked rs190509934) associated with reduced ACE2 expression and reduced likelihood of infection with SARS-CoV-2 (30, 31). Likewise, increased susceptibility to infection is associated with *SLC6A20* on chromosome 3p21.1, which encodes a protein [sodium-imino acid transporter 1 (SIT1)] that is associated with ACE2 (30, 31). Dipeptidyl peptidase-4 (DPP-4) has been identified as another possible candidate for SARS-CoV-2 binding *in silico*, albeit of unclear clinical significance (32–36). In addition to TMPRSS2, proteolysis can also be performed by endosomal cathepsin B (catB) and cathepsin L (catL) (23). Following fusion, the release and subsequent translation of ORF1a and ORF1b lead to the production of polypeptides pp1a and pp1ab, which are eventually processed into 16 non-structural proteins that comprise the viral RTC (23) (**Figures 1A, B**). Structural proteins are encoded from interspersed ORFs at the 3’ end of the ssRNA and are eventually processed in the endoplasmic reticulum and Golgi apparatus with modifications including N- and O-glycosylation, which are thought to be critical to virion infectivity (23, 37). Among the proteins produced during acute infection are replicase proteins, which help form replication complexes within the endoplasmic reticulum (38).

One mechanism of protection from pattern recognition receptors (PRRs), such as melanoma differentiation-associated protein 5 (MDA5), includes the formation of double-membrane vesicles (38). Relatively unchecked viral replication and an immune response that can be in disarray in severe disease can lead to the eventual dissemination through deeper inhalation of upper respiratory virus-containing particles or contiguous spread from the site of infection (38). Further dissemination of SARS-CoV-2 to other organ systems can be mediated through viremia, which is associated with adverse clinical outcomes (39). SARS-CoV-2 can evolve within a host to develop enhanced infectivity, immune evasion, and transmissibility, all of which can translate to pathogenicity and disease severity on the individual and population levels (40). A mutation rate of approximately 1 × 10^–6^ to 2 × 10^–6^ mutations per nucleotide per replication cycle is one characteristic of SARS-CoV-2 evolution (40). Selection pressures such as innate immune defense mechanisms like those mediated by the apolipoprotein B mRNA editing enzyme, catalytic polypeptide-like (APOBEC) family provoke the formation of C->U mutations, which can alter phenotypic properties of the virus ￼, which can, in turn, lead to the development of variants of concern (VOC). Transmissibility resulting from molecular evolution represents a bottleneck for mutations to pass from one host to another and in turn the likelihood of a virus like SARS-CoV-2 to cause an epidemic or pandemic (40). As a virus enters and uses the host cell for its own propagation, a response is elicited in the human host to fight for its own preservation. In the subsequent sections, that response—characterized by distinct but often overlapping cellular and extracellular mechanisms that accompany each stage of infection with SARS-CoV-2—will be elucidated.

## 2 Acute immunologic and inflammatory response to SARS-CoV-2

### 2.1 Innate immune responses to acute infection with SARS-CoV-2

The innate immune response to SARS-CoV-2 is the first line of defense against the virus (41) and also represents a crossroad at which immune cell recruitment and coordination can be pathologically skewed. Inflammatory cytokine disarray with concomitantly delayed IFN-I and IFN-III responses may be primary mediators of immune dysregulation in severe disease (42). Dysregulated innate immune responses comprise one of the critical features of COVID-19 (42).

#### 2.1.1 Host cell viral recognition

Early pattern recognition receptor and Toll-like receptor activation in SARS-CoV-2.

In normal physiologic conditions, the entry of viral RNA into a host cell does not go unnoticed. The newly released viral ssRNA contains pathogen-associated molecular patterns (PAMPs) recognized as foreign entities by pattern recognition receptors (PRRs) in the cytoplasm and along the cell membrane and endosomal surfaces (43–45). PRRs include cytoplasmic retinoic acid-inducible gene I-like receptors (RLRs) and membrane-bound Toll-like receptors (TLRs), with subclasses that recognize a wide array of viral nucleic acid assortments (44, 45). As a positive-sense ssRNA, the viral RNA from SARS-CoV-2 is recognized by the RLRs retinoic acid-inducible gene (RIG-I) and MDA5 by its uncapped 5’ triphosphate terminus (43). The activation of RIG-I and MDA5 and their association with mitochondrial antiviral signaling protein (MAVS) eventually leads to the transcription of type I interferons, which are likely dependent on nuclear factor kappa-light-chain enhancer of activated B cells (NF-κB) (45).

Positive-sense ssRNA also activates endosomal TLR7 (as well as TLR8), which, in conjunction with myeloid differentiation primary-response gene 88 (MyD88), typically leads to the production of both type I and type III interferons (IFNs) (46, 47). The TLR7/MyD88 pathway mediates immunity in plasmacytoid dendritic cells (pDCs) in multiple viral infections and is also likely relevant in airway epithelial cells infected with SARS-CoV-2 (48, 49). Furthermore, the severity of illness in young male patients with loss-of-function variants in the X-linked *TLR7*, as well as the associated defects in type I and type II interferon production, suggest a critical role of this pathway in the innate response to SARS-CoV-2 infection (46). Endosomal TLR3 may also be activated in the presence of SARS-CoV-2, perhaps due to transient dsRNA produced in the viral replication process (43, 49). TLR3 binds to TIR-domain-containing adapter-inducing interferon-β (TRIF), which also can lead to type I and type III interferon production via IFN regulatory factor 3 (IRF3) as well as robust NF-κB activation (43, 45, 50). MyD88 activation can polarize macrophages towards a pro-inflammatory M1 phenotype in mouse models (51). In addition to the above mechanisms, human airway epithelial cells infected with SARS-CoV-2 have also been shown to have decreased transcription of dual-specificity phosphatase 1 and 5 (*DUSP1* and *DUSP5*), which may lead to unbalanced activation of NF-κB (52).

#### 2.1.2 Compounded inflammation in acute SARS-CoV-2 infection at the level of the host cell

SARS-CoV-2 provokes the release of pro-inflammatory cytokines via NF-κB, inflammasome production, and pyroptosis.

Cytopathic viral infection of airway epithelial cells can trigger inflammasome activation, pyroptosis, necroptosis, and apoptosis (28, 53–55). Early studies during the pandemic revealed intense NLRP3 inflammasome expression in the lungs of patients with fatal COVID-19 (56), suggesting a pivotal role for pyroptosis in severe COVID-19 (57). SARS-CoV-2 may promote pyroptosis through non-structural protein 6 (nsp6) (58). The subsequent release of damage-associated molecular patterns (DAMPs) and activation of IL-1β may be one of the first events leading to the inflammatory cascade that characterizes COVID-19 (28). The release of DAMPs and IL-1β promotes the release of additional inflammatory cytokines such as macrophage inflammatory protein 1α (MIP1α), MIP1β, IL-6, and interferon gamma-induced protein 10 (IP-10) from adjacent alveolar macrophages, epithelial cells, and endothelial cells (28, 59). TGF-β production may also be enhanced in severe disease (60).

An essential function of ACE2 is to degrade angiotensin II (Ang II) into Ang- (1, 7, 61). As SARS-CoV-2 binds to ACE2 and the complex is endocytosed or incorporated through other mechanisms (62), less ACE2 is available to perform its typical functions, and levels of Ang II rise. The increasing levels of Ang II provide excess stimulation to the Ang II Type 1 receptor (AT1R), which—in addition to vasoconstriction—promotes the activation of NF-ĸβ through ADAM17 cleavage of TNFα, membrane-bound EGF (63), and IL-6Rα into their soluble forms (61). Subsequent activation of TNFαR and EGFR also leads to downstream NF-κB activation (61, 63). The activation of gp130 by the IL6R-α-IL-6 complex further activates a JAK/STAT pathway, amplifying the NF-κB response via STAT3 (63).

NF-κB plays a pivotal role in the upregulation of the immune response (64). It has been hypothesized that the benefits of corticosteroids such as dexamethasone may be partially due to the downregulation of NF-κB pathways (65). In airway epithelial cells, NF-κB promotes the production of IL-6, G-CSF, GM-CSF, and MIP2 kβ (66). In M1 alveolar macrophages, NF-κB promotes the production of an array of inflammatory cytokines, including IL-1β, IL-6, IL-12, and TNFα (64). NF-κB in SARS-CoV-2-infected hosts has also been shown to trigger NLRP3 (57) inflammasome formation and pyroptosis in monocytes through a caspase-1 mechanism with resultant production of DAMPs and cytokines such as IL-1β (67, 68). DAMP-mediated signaling itself leads to a conformational change in NLRP3 and caspase-1-mediated cleavage of the inhibitory C-terminus of gasdermin D, which is the main effector of pyroptosis (57). Gasdermin D can then traffic to the cell membrane and polymerize into β-barrel pores, promoting ion (namely, Ca2+) and protein flux and the eventual loss of the mitochondrial membrane potential and IL-1 and IL-18 activation and IL-1β release (57).

Other cytokines include IL-2, IL-17, IL-8, macrophage inflammatory protein 1 (MIP-1), monocyte chemoattractant protein 1 (MCP-1), and chemokine ligands (e.g. CCL2, CCL3, and CCL7) including C-X-C motif chemokine ligands (e.g. CXCL2 and CXCL10) (65). The clinical significance of cytokine release was demonstrated in a 2021 single-cell and bulk RNA-seq analysis of specimens from patients with COVID-19, which showed that upregulation of IL-2, IL-6, IL-8, IL-17A, and NF-κB was correlated with the severity of COVID-19 (69). The function of several such cytokines and chemokines in COVID-19 are summarized in **Table 1**. The release of cytokines in COVID-19 is associated with a hyperinflammatory state, leading to what some have called a “cytokine storm” that characterizes severe COVID-19 and its associated end-organ dysfunction syndromes (61, 63), similar to that seen in other hyperimmune responses characterized by cytokine release syndromes (CRS) related to chimeric antigen receptor-modified T (CAR-T) cell therapy, macrophage activation syndrome (MAS), and hemophagocytic lymphohistiocytosis (HLH) (70, 71).

**Table 1:** Cytokine and chemokine response in COVID-19.

Cytokine/Chemokine	Function
IL-1β	Proinflammatory (383); evidence of inflammasome activation (384); may induce ACE2 shedding (383)
IL-1RA	Evidence of inflammasome activation (99)
IL-2	Upregulation correlates with severity of COVID-19 (69)
IL-6	Levels highly correlated with SARS-CoV-2 RNAemia (385) and COVID-19 severity (69, 386); differentiation of monocytes to macrophages (70); mediates differentiation of T_H_17-cells (60, 153, 154); correlates with increase in DN2 B-cell population (181); persistent elevation (at least 8 months following infection) may be seen in PASC (301)
IL-7	Elevated in COVID-19 (387); important in T-cell development and survival (387)
IL-8	Proinflammatory via neutrophil recruitment (388); upregulation correlates with severity of COVID-19 in some studies (69, 388); may be associated with duration with illness (388); may be elevated in PASC (389)
IL-10	Key anti-inflammatory mediator which may also blunt anti-SARS-CoV-2 response (386); associated with COVID-19 severity (386)
IL-17	Pro-inflammatory; associated with neutrophil attraction (311); upregulation of IL-17A correlates with severity of COVID-19 (69)
IL-18	Evidence of inflammasome activation (99, 384); induces IFN-γ production (384); high levels seen in MAS, of note (384)
IL-21	Released by T_H_17 (155); may interact with IFN-γ to facilitate DN2 B-cell differentiation (181)
IL-22	Released by T_H_1, T_H_17 (155, 390), and NK cells (390); anti-inflammatory (390), anti-apoptotic, and anti-oxidant properties specifically on epithelial cells and fibroblasts (391)
IFN-α (T1 IFN)	Critical component of antiviral response (392); significant impairment (low levels) in severe and critical COVID-19 (94, 393); IgG autoantibodies to IFN-α2 (285, 309) may be seen in patients with critical COVID-19 (309) which may persist for at least 2-3 months after infection (285)
IFN-β (T1 IFN)	Critical component of antiviral response (392); significant impairment [absent, in at least one study (94)] in severe and critical COVID-19 (94, 393); persistent elevation (at least 8 months following infection) may be seen in PASC (301)
IFN-γ (T2 IFN)	May be a key component of cytokine-mediated inflammatory cell death and shock state together with TNFα (394); Drives extrafollicular B cells to antiviral antibody production (181, 395); persistent elevation (at least 8 months following infection) may be seen in PASC (301)
TGF-β	Induces peripheral plasmablast antibody production (namely, IgG1 and IgA1) (396) mediates differentiation of T_H_17-cells (60, 153, 154); may inopportunely suppress NK cell function in severe COVID-19 (397)
TNFα	Proinflammatory (383); may induce ACE2 shedding (383); may be a key component of cytokine-mediated inflammatory cell death and shock state (394)
CCL2/MCP-1	Elevated in COVID-19 patients compared to matched healthy controls (311); may indicate monocyte chemoattraction to affected lung (94)
CCL3/MIP1α	Elevated in COVID-19 patients compared to matched healthy controls (311)
CCL4/MIP1β	Elevated in COVID-19 patients compared to matched healthy controls (311)
CXCL9	IFN-γ-induced (384); may be elevated in cytokine storm syndromes (384)
CXCL10/IP-10	Correlates with disease progression (82) and an increase in DN2 B-cell population (181); may remain elevated throughout viral infection, which may be unique to severe coronavirus infections (82); associated with an increase in EF B cells (181)

Hyperinflammatory immune responses are not necessarily unique to SARS-CoV-2 (72, 73): High levels of IL-6 have been noted in patients with acute respiratory distress (ARDS) before the COVID-19 pandemic (74). Others have suggested that lymphocyte depletion and exhaustion, delayed interferon response, and decreased TNFα production in certain phases of the disease are indicators of immune suppression (75). Although a more precise depiction of the cytokine-mediated inflammatory response in COVID-19 may be suggested by the term “cytokine disharmony” or other terms denoting dysregulation, by and large, the immune response in severe forms of COVID-19 appears to skew towards a hyperinflammatory cytokine dysregulation (76–81), with early outcomes in severe disease perhaps attributable to cytokines preferentially elevated in SARS-CoV-2 (73, 82), interferon I dysregulation (83), and local microthrombi formation (74, 84), as will be discussed in further detail.

#### 2.1.3 The role of interferons

Interferon release is suppressed early in the course of SARS-CoV-2 infection.

Interferons are integral to the antiviral immune armamentarium and have been shown to have potent anti-SARS-CoV-2 activity (85). Critical illness in COVID-19 is associated with inborn errors of genes involved in Type I interferon production (86). While IFN-α and IFN-β can contribute to inflammation in SARS-CoV-2 through IFNR-mediated downstream activation of NF-κB, the simultaneous stimulation of interferon-stimulated genes (ISGs) can promote the development of a potent antiviral state (87, 88). First, IFN acts to increase PRRs, which can improve viral detection (88). In tandem, many proteins are produced via ISGs, which can inhibit nearly every step in viral infection and replication (88).

Despite their seemingly potent anti-SARS-CoV-2 activity, the transcription of crucial interferon genes for type I and type III interferons (*IFNB, IFNK, IFNA5*, and *IFNL1-5*) is suppressed early in the course of SARS-CoV-2 infection, particularly in severe disease (42, 89). Mechanisms may include suppression of sequential steps along the pathway of ISG production, similar to that mediated by other coronaviruses such as SARS-CoV-1 [mediated in part by Nsp1 (90)] and MERS-CoV in humans and murine hepatitis virus (MHV) in mice (91–93). This phenomenon, among others, may help to explain the pathogenicity and associated immune dysregulation of SARS-CoV-2 (85, 89, 94, 95).

#### 2.1.4 Soluble pattern recognition molecules

Soluble pattern recognition molecules (PRMs) have antibody-like functions representing a key component of humoral immunity (96). These include collectins (e.g., mannose-binding lectin (MBL), which activates the complement system via the lectin pathway), pentraxins [e.g., C-reactive protein (CRP)], C1q, and ficolins (96). Systemic complement activation was upregulated in patients with COVID-19, with sC5b-9 and C4d found to be higher in those patients with respiratory failure (97). A 2022 study revealed that MBL and pentraxin 3 (PTX3) play critical roles in humoral immunity against SARS-CoV-2, with MBL binding to spike (S) protein in a glycan-dependent manner prompting complement activation via the lectin pathway and with PTX3 binding to nucleocapsid (96) (**Figure 1J**). Furthermore, single nucleotide polymorphisms (SNPs) of the *MBL2* gene, particularly rs10824845, suggest an association with the severity of COVID-19 (96). Interestingly, despite its apparent association with clinical outcomes, CRP was not shown to bind to any of the SARS-CoV-2 proteins tested, which included S, N, and E (96, 98).

#### 2.1.5 Monocytes and macrophages

Monocytes and macrophages play a prominent role in the dysregulated immune response to SARS-CoV-2 (71). Macrophages express ACE2 receptors and represent targets for SARS-CoV-2 viral entry [which may also occur via the Fc receptor CD16 (99)], viral replication (99) and spread (100, 101), and associated immune responses (102). Responsiveness to TLR associated with suppressing IL-1R-associated kinase (IRAK)-M expression points to hyperinflammatory phenotype (102). Macrophages mediate cytokine release in COVID-19 through STAT1 signaling and pDC activation and may be associated with a dampened IFN-I response (103, 104). Once activated during SARS-CoV-2 infection, macrophages showed signs of inflammasome activation as evidenced by an associated speck-like protein containing a CARD (ASC) co-localization with NLR family pyrin domain containing 3 (NLRP3) and activated caspase-1, as shown in a recently published *in vivo* murine model with a humanized immune system (99). IL-18 and IL-1RA (considered downstream evidence of inflammasome activation) were upregulated (with IL-1β also evident *in vitro*), as was chemokine CXCL10 expression (99). Finally, pyroptosis was also increased in this model as measured by LDH and gasdermin D levels (99). Similar results were seen in *in vitro* studies of monocytes (67). IL-6 further promotes the differentiation of monocytes to macrophages (70). Perhaps owing to the ubiquity of macrophages in tissues (105), macrophage-induced inflammation—mediated in part by direct viral infection, inflammasome activation, pyroptosis (57), and cytokine release (70, 103, 104)—appears to be a prominent driver of the SARS-CoV-2-mediated immune response (101, 106) (**Figures 1C–F**).

#### 2.1.6 Plasmacytoid dendritic cells

Plasmacytoid dendritic cells (pDCs) help drive the immune response to viral infections such as coronaviruses (107). pDCs are more likely to be directly infected with SARS-CoV-2 than macrophages (104), and emerging evidence has shown that pDCs are the predominant cell type involved in the production of Type I and Type III interferons (108). COVID-19 severity is inversely correlated with a pDC-driven IFNα response (108). The presence of pDCs was seen in the lungs of patients with SARS-CoV-2 and was associated with IFN-I signaling, itself correlated with macrophage inflammatory response (104). pDCs have been shown to enhance TLR signaling in macrophages via IFN-1; RNAseq studies have additionally shown mediation of transcriptional changes in macrophages that correlate with a more robust inflammatory response to SARS-CoV-2 than to LPS (104). A 2021 study showed that both myeloid and plasmacytoid dendritic cells show an alteration in DC homing and activation markers such as PDL-1, CD86, and CCR7 that correlated with hospitalization status (109). Given the central importance of macrophages in COVID-19, the mechanistic pathways involving pDCs are likely a critical first step in developing the hyperinflammatory cytokine response accompanying macrophage activation in severe disease. Functional alterations of DCs and deficiency of pDCs (as well as CD1c+ mDCs) may persist seven months after acute SARS-CoV-2 infection (109).

#### 2.1.7 Neutrophils

In addition to their overt role in bacterial and fungal infections, neutrophils can contribute to the immune response to viruses (110). They may additionally exhibit enhanced trafficking to highly vascularized organs such as the lungs and kidneys (111). Neutrophilia (112) and a high neutrophil-to-lymphocyte ratio are associated with severe COVID-19 (113, 114). Early studies using single-cell transcriptomics and proteomics from peripheral blood mononuclear cells (PBMC) of patients with COVID-19 showed altered myelopoiesis in patients with severe disease (115). Immature neutrophils expressing CD24, DEFA3, DEFA4, and PGLYRP1 were a prominent cell population in that study (115). Another study showed a reduction in the neutrophil maturation marker CD-10, associated with emergency myelopoiesis (400) and poor clinical outcomes (116). Among the mechanisms studied, the formation of neutrophil extracellular traps (NETs) (aggregates of extracellular DNA, histones, neutrophil elastase, myeloperoxidase, and other molecules such as tissue factor) (111) are thought to play a prominent role in COVID-19 pathogenesis, mediated in part through target-organ microvascular occlusion (111) and associated immunothrombosis (111, 117) (**Figure 1I**).

#### 2.1.8 Natural killer cells

Acknowledging the innate-adaptive overlap exhibited by natural killer (NK) cells (118), NK cells play an essential role in innate antiviral responses (119, 120). While an increased frequency of activated NKG2C^+^CD57^+^ CD56^dim^ NK cell phenotypes may be seen in severe COVID-19 (121), severe infection is associated with peripheral NK cell depletion and an exhausted phenotype based on LAG3, PDCD1, and HAVCR2 expression, which may be mediated by aberrant TGF-β production and homing to lung tissue (121, 122). NK cell quiescence manifesting as decreased perforin and granzyme production has also been demonstrated in COVID-19. This may be mediated by increased IL-6 (123) and TNFα, both part of the cytokine milieu characterizing the syndrome (124).

#### 2.1.9 Innate lymphoid cells

Innate lymphoid cells (ILC) are more recently described subsets of lymphocytes that can participate in innate immune responses that mirror type 1, type 2, and type 3 immune responses (125). Studies have shown that illness severity in COVID-19 is inversely associated with ILC count (126, 127). ILC-mediated amphiregulin production has been proposed as one mechanism of immune tolerance (126). Of note, a proportionally higher number of amphiregulin-producing ILC was seen in healthy controls vs patients hospitalized with COVID-19 and in females versus males (126).

#### 2.1.10 Mucosal-associated invariant T-cells

Mucosal-associated invariant T-cells (MAIT) are innate-like T cells that recognize non-specific riboflavin metabolites of bacteria at mucosal surfaces, including those lining the bronchial tree, which are also activated during viral infections (128, 129)and can inhibit viral replication (130). While the specificity and significance of this cell type in COVID-19 is still under study, heightened activation of circulating CD8+ MAIT cells (127, 128) may correlate with illness severity as measured by Simplified Acute Physiology (SAPS) II score (128).

#### 2.1.11 γδ-T-cells

γδ-T-cells are another subset of T-lymphocytes with innate-like functionality with the potential to induce strong antiviral responses independent of MHC-mediated antigen presentation (122, 131, 132). Their use in cancer immunotherapy (122, 133) and presence at mucosal barrier sites (134) stimulated interest in their role in COVID-19 (133, 134). In SARS-CoV-1, they appear to be active members of the immune milieu (133, 135). However, their role in SARS-CoV-2 appears to be muted (131) compared to SARS-CoV-1^120^. In addition to their peripheral depletion, as with other T-cell subsets (136), γδ-T cells could not mount a potent immune response to spike or nucleocapsid antigen *in vitro* studies (131). The depletion of various innate-like lymphocyte subtypes is portrayed as a component of the early immune response to SARS-CoV-2 in **Figure 1K**.

### 2.2 Adaptive immune responses to SARS-CoV-2 infection

Components of the innate immune response to SARS-CoV-2 infection can help in the immediacy of their response while simultaneously perpetuating detrimental non-specific inflammatory signaling. The adaptive immune response generally follows the innate immune response and provides an additional layer of nuanced and potentially potent protection in patients with and recovering from COVID-19. In addition, the memory adaptive response induced by both natural infection and vaccination, as discussed below, plays a crucial role in protection from reinfection, limiting disease severity and hastening resolution in the event of reinfection.

**Figure 2:**
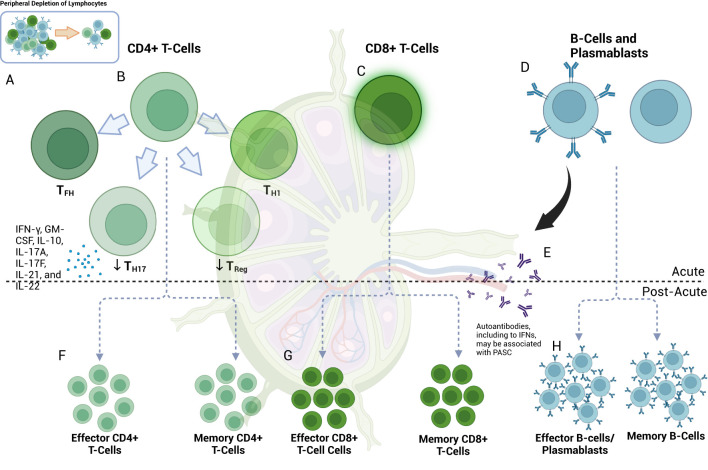
Lymphocyte response in acute and post-acute SARS-COV-2. **(A)** Lymphopenia is a hallmark of COVID-19 (408). **(B)** Among the cell types present, CD4+ T-cells skew towards T_H1_ and T_FH_ phenotypes with associated effector functions (145, 151); germinal center T_FH_ may be decreased (151). Upregulation of T_H_17-cells can occur [in contrast to T_reg_ (156)] and may be accompanied by the pro-inflammatory release of IFN-γ, GM-CSF, IL-10, IL-17A, IL-17F, IL-21, and IL-22 (155). **(C)** CD8+ cells exhibit activated phenotypes with preserved effector function and a potential role in macrophage-mediated cytokine release in acute COVID-19 (137, 161, 163) despite showing peripheral depletion (139, 144, 159, 160). **(D)** B-cells are also peripherally depleted in COVID-19 (173, 181). Plasmablast development occurs at first in extrafollicular zones (173), initially with less sophisticated targeting (173, 181) via DN2 and DN3 B-cells (181), but later [at approximately seven days post-infection (181)] with more robust class-switching, somatic hypermutation and affinity maturation (173) from germinal center (GC) cells. **(E)** Autoantibody development, including those directed towards interferons, can develop at this time (309) and may later be associated with PASC (285). **(F)** In the post-acute phase, effector CD4+ T-cell subtypes may remain active for at least 2-3 months (285). Decreased levels of naïve CD4+ T-cells suggest ongoing activation (301). Memory T-cells (which can develop early in the course of infection) can include those of the CD4+ and CD8+ T-cells and subtypes (165, 406), and may be detected at least one year post-infection (165). S-specific CXCR3-/CXCR5-/CCR6+ T_H_17-cells can have a half-life of 4.9 years (405). Memory T-cells may be important drivers of vaccine-mediated immunity (194, 195). **(G)** CD8+ T-cells may continue to exhibit markers of activation and exhaustion (PD-1 and TIM-3) at three months, particularly in patients with PASC, and have been observed to persist for up to 8 months (301). Memory CD8+ T-cells may have a half-life of one year (405); this can vary by disease severity (405). **(H)** Depletion of naïve B-cells suggests ongoing activation at eight months (301). Memory B-cell (MBC) frequency may correlate with decreased symptom duration (403) and may also have a role in vaccine-mediate immunity, even as antibody levels subside (192). MBC associated with autoantibody or SARS-CoV-2-directed antibody production may also be associated with varying PASC symptomatology (285). Of note, autoantibody production may occur early and may precede COVID-19 diagnosis (285).

The adaptive immune response plays an important role in viral infections, primarily consisting of CD4+ T-cells, CD8+ T-cells, and B-cells (137). Despite this role, lymphopenia is a hallmark of COVID-19 and may correlate with disease severity and severe lung injury (127, 138, 139) (**Figure 2A**). In addition to the direct cytopathic effects of SARS-CoV-2 (140), early studies showed significant damage to lymphoid tissue and lymphocyte apoptosis in the lymph nodes and spleen of deceased patients with COVID-19 (141). Additional mechanisms may involve sequestration or recruitment of lymphocytes to actively inflamed tissues such as the lung and other target organs (127, 142). Lymphopenia also appears to be inversely related to levels of IL-6 and therapeutically targeted IL-6 inhibition can lead to the correction of circulating lymphocyte counts (143, 144).

#### 2.2.1 CD4+ T-cells

CD4+ T-cells are integral to developing a targeted adaptive immune response by recognizing foreign antigens on MHC Class II molecules and activating other immune cells, including CD8+ T-cells, B-cells, and NK cells (145). Specific viral targets of CD4+ T-cells include M, N, and S, nsp3 and ORF3, and others, albeit to a smaller degree (137, 146). Tan et al. and others showed that a robust CD4+ T-cell response is important for viral clearance in SARS-CoV-2 (147) and that the relative absence of such a response could be fatal (148). Early studies showed a more prominent CD4+ T-cell response than CD8+ T-cells (137), with increased expression of CD38 in moderate-to-severe disease indicative of an activated phenotype (149). An increased proportion of cytotoxic CD4+ T helper (CTL-T_H_) cells and follicular T_FH_-cells (which have a role in B-cell affinity maturation and antibody production) were seen in hospitalized patients with COVID-19 relative to regulatory T-cell (T_reg_) populations (145). Curiously, another study showed a positive correlation of specifically CD8+ T_reg_ cells with increased CD4+ cells in patients with acute SARS-CoV-2 infection (150), a finding potentially indicative of a compensatory anti-inflammatory program (89).

As noted, depletion of T-cells, particularly in the peripheral blood, is a hallmark of severe COVID-19 (144, 149). T-cells exhibit enhanced expression of apoptotic pathways and T-cell immunoglobulin and mucin-domain containing-3 (Tim-3) and programmed cell death protein 1 (PD-1), which—while expressed in activated states—can also be markers of T-cell exhaustion (139). In addition, germinal centers—which are important sites of T-cell and B-cell interaction—are lost in thoracic lymph nodes of patients with acute COVID-19, with an associated loss of germinal center CD4+ Bcl-6+ T_FH_-cells and an increased proportion of T_H1_-cells (151), which are responsible for IFNγ production. Bcl-6 is a transcriptional repressor that can allow for the development of high-affinity immunoglobulins in both T and B-cells (152). Taken together, T-cell lymphopenia, exhaustion, and associated immune sequelae likely contribute to the immune dysregulation in COVID-19 (**Figure 2B**).

#### 2.2.2 T_H_17 cells

T_H_17 cells are a distinct lineage of CD4+ T-cells that are typically involved in the clearance of extracellular pathogens (153). Their differentiation is mediated through TGF-β and IL-6 (153), both of which are upregulated in severe COVID-19 (60, 154). T_H_17 cells may contribute to acute inflammation in COVID-19 through the release of IFN-γ, GM-CSF, IL-10, IL-17A, IL-17F, IL-21, and IL-22 (155) (**Figure 2B**). An increased T_H_17/T_reg_ ratio and their associated factors (e.g., RAR-related orphan receptor gamma (RORγt)/Forkhead box protein P3 (FoxP3) appear to correlate with SARS-CoV-2 infection as well as clinical outcome (156). As will be discussed, T_H_17 cells have been implicated in chronic autoimmune and inflammatory conditions and thus may represent an important link between acute and chronic immunity in patients with COVID-19 (153).

#### 2.2.3 CD8+ T-cells

CD8+ T-cells target cells infected with intracellular pathogens, including viruses such as SARS-CoV-2, by detecting foreign antigens on MHC-1 (137, 157). CD8+ T-cells can target numerous proteins derived from SARS-CoV-2, depending on expressed HLA class I types (146). While widely viewed as critical for disease resolution and subsequent protection from re-infection (137), SARS-CoV-2-specific CD8+ T cell responses in the blood tend to be relatively low in frequency (compared to influenza or Epstein-Barr virus) in most infected or resolved individuals (158). Several studies have confirmed a decrease in circulating CD8+ T-cells in COVID-19 (139, 144, 159, 160). Those CD8+ T-cells that were present were found to be highly activated in a subset of COVID-19 patients as measured by KI67 and HLA-DR+CD38 expression (161) and appear to retain their cytotoxic capacity as measured by IFNγ, granzyme B, perforin, and CD107a production or expression (137) (**Figure 2C**). In addition, pre-existing cross-reactive nucleocapsid-specific memory CD8+ T-cells that recognize seasonal coronaviruses (162) may provide some level of protection from primary SARS-CoV-2 infection or disease severity, depending on the specificity and HLA haplotype. CD8+ T-cells have also been implicated in non-COVID cytokine release syndromes such as macrophage activation syndrome (MAS) (163) and may play a similar role in the exacerbated immune response in COVID-19.

#### 2.2.4 Memory T-cells

Memory T-cells can recognize and aid in clearing viruses to which a host may be re-exposed (164). Given the novelty of SARS-CoV-2, the duration of detectable memory T-cells responsive to SARS-CoV-2 remains the subject of ongoing study. Highly reactive, polyclonal CD8+ T-cells have been detected in the serum of patients with a history of mild COVID-19 up to one year after infection (165), with recent studies supporting at least this duration for SARS-CoV-2-specific CD8+ T-cells expressing CD45RA, IL-7R-α, T cell factor 1, and low CCR7 (166). This phenotype suggests tissue recirculating effector memory CD8+ T cells that retain high proliferative potential yet traffic through lymphoid and non-lymphoid tissues (167). Of note, memory T-cells have been detected in patients with SARS-CoV-1 beyond 17 years (164). Importantly, SARS-CoV-2-specific stem-cell-like memory T-cell populations appear to be maintained regardless of disease severity (164) (**Figures 2F, G**). While the clinical significance of memory cell phenotypes remains to be determined, particularly in the context of viral variants (89), evidence suggests that CD8+ T-cells generated through natural immunity (168) or vaccination (169) can cross-recognize viral antigens despite a significant number of mutations in the viral genome (169).

#### 2.2.5 B-cell-mediated immune response: humoral immunity

B-lymphocytes are a critical component of adaptive immunity. Antibodies secreted by B-cell-derived plasma cells can serve multiple roles through antigen binding: Neutralization by preventing pathogen entry, immune cell recruitment and/or activation, and enhancement of antigen uptake and processing by antigen-presenting cells to other immune cells (170). Antibodies have also historically provided a measurable immune response that can serve as an imperfect but established benchmark for immunity (170–172).

#### 2.2.6 Naturally occurring antibodies

Pre-existing antibodies to SARS-CoV-2 in humans were predominantly non-neutralizing, directed towards the N-protein and, to a lesser extent, the largely conserved S2 component of the S protein, which does not contain the receptor binding domain (RBD) (173, 174), with the latter suggesting some degree of cross-reactivity from endemic coronaviruses (174, 175) (**Figure 1G**). Anti-glycan antibodies—by and large of the IgM class (176)—may also be found in humans at baseline and can be targeted towards glycans found on enveloped viruses (176). Lower risk of COVID-19 in patients with blood type O (177) may have contributed to the hypothesis that certain blood types may be more protective against SARS-CoV-2 infection, perhaps by anti-glycan ABO antibodies (177). Early GWAS of COVID-19 patients provided a biologically plausible mechanism for this proposed phenomenon by identifying a reproducible association of susceptibility to COVID-19 with locus 9q34.2, which coincides with the ABO locus (178, 179). However, observational data since the discovery of the virus have been mixed (176, 180), perhaps owing to the difference between susceptibility and clinical severity of the disease (179).

#### 2.2.7 Acute B-cell response to SARS-CoV-2 Infection

The acute phase of SARS-CoV-2 infection is accompanied by B-cell lymphopenia and early plasmablast development from extrafollicular sites, a response typically occurring within 3-5 days (173, 181). In contrast to other infections, early antibody responses to SARS-CoV-2 include IgG, IgA, and IgM production (173) directed predominantly toward the N and S proteins (173). Somatic hypermutation and affinity maturation are less likely to occur at this stage (173, 181), and plasma cells produced during this time tend to be short-lived (173). The degree of extrafollicular B cell activation is characterized by an increase in CD11c^+^ activated naive (aN) B-cells which are precursors to IgD^-^CD27^-^, C-X-C chemokine receptor type 5 (CXCR5)^-^CD21^-^ [double-negative 2 (DN2)] B-cells in addition to more recently discovered Cd11^-^CD21^-^ (DN3) B-cell populations in severe COVID-19, which correlates with an increase in antibody-secreting cell populations (181) (**Figure 2D**). The differentiation of these cells is TLR7-independent and is mediated through IFNγ-IL-21 interactions, with the increase in DN2 B-cells additionally found to correlate with levels of IL-6 and IP-10 (181). Notably, an increase in endogenous neutralizing antibodies correlates with severe disease (181) and thus may suggest a raging battle rather than a victorious immune response. Curiously, a similar B-cell activation phenotype has been found in active SLE (181).

Approximately seven days after infection (181), a more nuanced and durable B-cell follicular response begins to develop from germinal center (GC) cells in lymphoid tissue via class-switching, affinity maturation, and somatic hypermutation (173). IgM and IgG anti-N, anti-S (182), and specifically anti-RBD (183)-defined seroconversion typically occur at a median of 11-13 days after symptom onset (173, 182, 183). In severe COVID-19, however, the GC response may be markedly blunted: Post-mortem specimens revealed an *absence* of germinal centers in the thoracic lymph nodes of patients with acute COVID-19 (151). A reduction of Bcl-6^+^ GC B-cells was seen in the same patient population (151). As previous research points to a quantitatively robust and seemingly adequate neutralizing capacity of the early extrafollicular antibody response (181), it is conceivable that the sustained production of meticulously targeted antibodies may be adversely impacted. However, the mechanism underlying this relationship likely requires further study (184).

#### 2.2.8 Memory B-cells

Memory B-cells (MBCs) provide an immune reservoir for clonal expansion after infection (185, 186). In acute SARS-CoV-2 infection, the extrafollicular B-cell response includes the development of transient non-class-switched MBC with low rates of somatic hypermutation and class-switching (173). In tandem, pre-existing MBC populations with cross-reactivity to conserved S2 regions can proliferate, as evidenced by a robust initial IgG response to S2 (174). B-cells that enter germinal centers of secondary lymphoid organs can develop a more nuanced immune memory through class switching and somatic hypermutation later in the course of infection (173). Patients who recover from COVID-19 have an increased proportion of tissue-like memory (TLM) IgG+ S1-specific MBC (187) and circulating resting IgDloCD20+ MBC sustained for at least seven months after acute infection (188) (**Figure 2H**). The process of MBC generation likely requires CD4+ T-cells and can be affected by the severity of illness (187). Among the various immunoglobulin subtypes, IgG MBC eventually predominates in recovered individuals, along with a minor population of IgA MBC (171, 173).

#### 2.2.9 Long-lived bone marrow plasma cells

Long-lived CD19^−^CD38^hi^CD138^+^ bone marrow plasma cells (BMPC) are another component of the humoral memory (173, 189) and may be the primary source of circulating antibodies years after a viral infection (188). Their presence has been detected at least seven months following infection with SARS-CoV-2 (188).

A summary of several important cell types and their function in acute and post-acute COVID-19 is shown in **Table 2**. Of note, while neutralizing antibody titers may suggest protective immunity early in the post-infectious period (172), the relative contributions of various immune memory cells to sterilizing or protective immunity in SARS-CoV-2 remain the subject of further study, making such immunity challenging to define at this time.

**Table 2:** Innate and adaptive immune responses to COVID-19.

	Cell Type or Molecular Immune Response	Expression and Function in Early COVID-19	Expression and Function in Post-Infectious COVID-19	
Innate/Humoral Immune Responses	NK cells	Depleted number with exhausted phenotype in COVID-19 (124, 398) despite antiviral potential (119, 120)	Memory-like NK cell at 2-3 months correlates with cough symptoms of PASC (285)	
	Innate Lymphoid Cells (ILC)	Depleted in severe illness (126, 127) in spite of amphiregulin-mediated immune tolerance potential (126)	Further research needed	
	MAIT	Peripherally depleted in severe illness (127); effector functions (primarily TNFα and IL-17A production) altered in COVID-19 (128) in one study, with heightened activation and cytotoxic phenotypes seen in another study (129) correlating with severity of disease	Cell levels normalize in convalescent phase albeit with suppression of CXCR3 levels, particularly in severe disease (399)	
	γδ-T-cells	-Peripherally depleted in COVID-19 (136) and unable to mount potent responses to spike and nucleocapsid antigens (131)	Innate-like CD3+CD4−CD8− T cells (which may include γδ-T-cells), found absent at 3 months in patients with PASC (301)	
	B-cells	Cross-reactive, naturally-occurring antibodies may confer some degree of protection (174–177); Peripherally depleted in COVID-19 (173, 181) albeit with activated responses (181); Early plasmablast development and antibody response in extrafollicular cells includes IgM, IgG and IgA (173) to N and S proteins (173) DN2 and DN3 cells characterize the EF response with adequate neutralizing capacity (181); Autoantibodies to type I and type III IFNs (e.g.) can be seen in COVID-19 patients (309, 415)	Autoantibodies may be seen in convalescence (285) and may play a role in PASC (285); Naïve CD127^low^TIM-3^−^CD38^low^CD27^−^IgD^+^ B-cell populations diminished in the post-acute phase, suggesting ongoing activation (301)	
	Monocytes/Macrophages	AACE2 and CD16–mediated viral infection (99, 102); Downregulation of IRAK-M expression (102) Inflammasome activation (99)	Monocytes continue to be polyfunctional throughout the convalescent phase (at least at 2-3 months) across multiple subgroups (285)	
	Plasmacytoid Dendritic Cells (pDCs)	Direct infection; production of Type I and Type III interferons (108); inverse correlation with COVID-19 severity (108); enhance TLR signaling in macrophages via IFN-1	Persistent reduction 7 months post hospitalization (109)	
	Neutrophils	Emergency myelopoiesis may produce immature subtypes (115, 400) with resultant pro-inflammatory and thrombogenic NET formation (111, 117); increased numbers and NLR may be associated with severe disease (113, 114)	Total neutrophil count and levels of MPO are increased in patients with post-COVID-19 interstitial lung abnormalities (401); NET formation may be associated with lung fibrogenesis (401); NETosis-associated immunothrombosis may be associated with long-term effects of COVID-19 (402)	
	Memory B-cells	May be source of initial IgG response in SARS-CoV-2 naïve patients by cross-reactivity to conserved antigens (174) Initially with low rates of somatic hypermutation and class-switching (173); Production likely requires CD4+ T-cells (187)	Increased frequency of MBC, IgM+, and class-switched MBC associated with decreased symptom duration in convalescent patients (403); likely role in vaccine-mediated immunity even as antibody levels decline (192)	
	BMPC	Likely source of circulating antibodies years after infection with other viruses (173, 189)	Detected at least 7 months after SARS-CoV-2 infection (188)	
	Soluble Pattern Recognition Molecules	Unclear for CRP (98) Systemic complement activation (97) MBL binds to spike protein (96); deficiency may be associated with severity (404)PTX3 binds to nucleocapsid (96)	MBL deficiency more frequent in PASC compared to historical controls (389)	
Adaptive/Cellular Immune Responses	CD4+ T-cells	Important role in viral clearance (147) with activated phenotypes (149); skew towards T_H1_ and T_FH_ phenotypes with associated effector functions (145, 151), albeit with quantitative loss of germinal center T_FH_-cells (151); Likely important in generation of MBC (187)	Naïve CD4+ cell populations diminished in the post-acute phase, suggesting ongoing activation (301); clonal expansion of cytotoxic CD4+ cells may continue 2-3 months following infection and may be associated with GI symptoms (285)	
	T_H_17 cells	May contribute to acute inflammation through release of IFN-γ, GM-CSF, IL-10, IL-17A, IL-17F, IL-21, and IL-22 (155); Appear to be proportionally upregulated in COVID-19 (as compared to T_reg_ (156), which may be associated with clinical outcome (156); may be a mechanism for COVID-19-related autoimmunity (155)	Half-life of S-specific T_H_17-like (CXCR3-/CXCR5-/CCR6+) cells is approximately 4.9 years, longer than T_H_1-, T_H2_-, and T_FH_-like cells (405); increase in proportion of T_H_17 central memory cells seen at least 24 weeks post-infection (406); noted role in autoimmune conditions like SLE (153)	
	CD8+ T-cells		Peripherally depleted in COVID-19 (139, 144, 159, 160) albeit with activated phenotype (161) and preserved effector function (137); possible role in macrophage activation-related cytokine release (163)	Naïve CD8+ cell populations diminished in the post-acute phase, suggesting ongoing activation (301); Clonal expansion of cytotoxic CD8+ cells may continue 2-3 months following infection and may be associated with GI symptoms (285)
	Memory T-cells	CD8+ T-cells present at least 1 year post-infection (165) and likely longer (164); may be an important driver of vaccine-mediated immunity (194, 195)	May the predominant mediator of immunity following decay of neutralizing antibody titers (407) and across pre-Omicron variants (407); S-specific CD8+ and CD4+ central and effector memory T-cells also shown to be likely predominant in vaccine-mediated immunity against Omicron variant (195); increase in proportion of CD4+ T_H_17 central memory cells seen at least 24 weeks post-infection (406)	
	Regulatory T-cells	Appear to be proportionally downregulated in COVID-19 (as compared to T_H_17 (156), which may be associated with clinical outcome (156)	Increase in TEMRA (effector memory with acquired CD45RA) Tregs at 16 weeks and naïve Treg to at least 24 weeks post infection with associated decreased in central memory, effector memory, T_H_R2, T_FH_R to 16 weeks and T_H_R22, T_H_R2/22 to 24 weeks post-infection (406)	

#### 2.2.10 Vaccine-mediated adaptive immunity

The severity of the COVID-19 pandemic spurred the development of highly effective vaccines with the benefit of low-risk immunity across a population. Currently, available SARS-CoV-2 vaccines contain mRNA (or DNA in adenovirus vaccines) encoding the S protein, which can be further cleaved into its subunits S1 and S2 (190). Initial studies with mRNA vaccines established immunogenicity based on clinical efficacy (6), IgG response to RBD or S1, and at least 50% neutralization titers (191). Later studies further showed the durability of vaccine-induced immune memory, as evidenced by an increase in CD71+ MBC cells 3-6 months post-vaccination, even as antibody levels to S and the RBD declined (192). Notably, MBCs were cross-reactive to alpha, beta, and delta variants per data available then (192). More recent findings highlight the effect of sequential vaccine boosters as promoting refined MBC antibody-mediated immunity that targets both the ACE2 binding site of SARS-CoV-2 spike protein as well as more conserved components of the RBD (i.e., Type 1/4 antibodies), which correlate with increasing potency of SARS-CoV-2 neutralization (185).

While not suited for neutralizing and preventing viral entry into host cells, T-cell responses can help prevent the spread of infection within a host and consequently can impact illness severity and virus transmissibility (193). Omicron variant vaccine efficacy has been shown to be adversely affected by Omicron-specific CD8+ T-cell responses despite moderate levels of neutralizing antibodies in macaques (194). More recent studies also suggest conserved vaccine-mediated cellular immunity across variants, including Omicron, despite a diminished neutralizing antibody response (195). While the authors would be remiss if we did not mention vaccine-mediated immunity, the topic itself warrants further exploration beyond this paper’s scope.

The immune response evoked by infection with SARS-CoV-2 at the molecular level is often accompanied by the clinical syndrome known as COVID-19. COVID-19 is widely recognized as a predominantly respiratory disease manifesting as fever and cough (196). It is also recognized that the virus and its inflammatory response can affect multiple organ systems outside of the respiratory tract, as described in the following section.

### 2.3 Acute immune-mediated end-organ injury following SARS-CoV-2 infection

**Figure 3:**
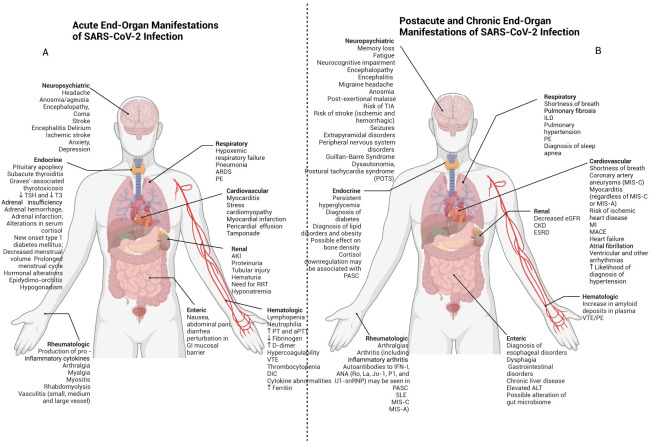
Acute, post-acute and chronic end organ manifestations of SARS-CoV-2 infection. SARS-CoV-2 may have adverse clinicopathophysiological impacts on multiple organ systems throughout the body. **(A)** Early effects are shown here and may include delirium, stroke, cardiomyopathy, myocardial infarction, pneumonia, ARDS, and AKI. **(B)** Postacute and chronic end-organ manifestations of SARS-CoV-2 infection can include neuropsychiatric symptoms such as fatigue and POTS (which may be features of PASC), in addition to myocarditis, interstitial lung disease and CKD.

Acute SARS-CoV-2 infection can lead to end organ damage through direct viral infection associated with localized immune activation and systemic immune responses. In addition to the lung, ACE2 receptors can be found in the heart (197), brain (198), kidney (199), testis (199), liver (200), colon (199, 201), and other organs (199, 202) (**Figure 3A**). While the clinical significance of such expression has not yet been fully elucidated, SARS-CoV-2 viremia can lead to end-organ infection and associated dysfunction by developing local pathologic inflammatory responses. Furthermore, the presence of ACE2 on endothelial cells (203, 204) and the known association with endotheliitis (197, 205) and microthrombi (203, 206) may allow for SARS-CoV-2 to affect multiple organs through disruption of the normal functioning of the microvasculature (197).

#### 2.3.1 Endothelial cell infection, microangiopathy, and microthrombi mediate end-organ dysfunction in COVID-19

ACE2 expression on endothelial cells may allow for viral infection to impact multiple organs, including the lung and kidney (207). Early pathologic specimens from COVID-19 patients revealed direct viral infection of endothelial cells in a transplanted kidney, with inflammatory lymphocytic infiltration noted in microvasculature of the heart, lung, kidney, and liver (207). While endotheliitis and hypercoagulation can be found with other viral infections, additional studies revealed the presence of intracapillary microthrombi in the pulmonary vasculature, as well as intussusceptive angiogenesis likely resulting from loss of normal microvascular architecture, which was seen more often in SARS-CoV-2 when compared to H1N1 influenza A (208, 209). Endothelial cell activation is associated with a hypercoagulable state in COVID-19 (210, 211), with proposed mechanisms including exocytosis of granules containing von Willebrand factor (vWF) and P-selectin (211) in addition to alteration in plasminogen activator inhibitor 1 (PAI-1) and tissue factor pathway inhibitor (TFPI) levels and upregulating cell adhesion molecules like VCAM and ICAM, which facilitates extravasation of circulating white blood cells (212) (**Figure 1H**).

The hypercoagulable state of COVID-19 may be a function of the complex interplay between immune cells and endothelial cells. Early polymorphonuclear (PMN) cell activation may lead to neutrophil extracellular traps (NETs) forming via NETosis, as suggested by increased plasma NET formation in intubated patients with COVID-19 (213). Colocalization of citrullinated histone H3+ neutrophils with platelets in blood vessels suggests that NET formation is implicated in the development of microthrombi in COVID-19 patients (213) (**Figure 1I**). Macrophages and monocytes in which inflammasomes and pyroptosis are activated may release microparticles containing tissue factor (TF) (214, 215), a critical first step in the coagulation cascade (216). IL-6 may further enhance the release of TF from monocytes (217). Of note, while not unique to COVID-19, severe infections can also promote the development of disseminated intravascular coagulation (DIC), the mechanisms of which have been well-studied (218).

#### 2.3.2 Acute lung injury

The lungs are among the earliest and most overtly affected organs in COVID-19 (219), with symptoms ranging from mild [and sometimes relatively asymptomatic (220, 221)] to severe hypoxemic respiratory failure requiring mechanical ventilation (222). Despite comparatively low ACE2 expression in the lung overall, type II alveolar epithelial cells (223) are preferentially targeted by SARS-CoV-2 (69). Early infection is typically characterized by viral pneumonia of varying severity (219, 224), which may progress to COVID-19-related acute respiratory distress syndrome (ARDS) (224) with a predominance of CD-3+ and CD-4+ T lymphocytes in precapillary and postcapillary blood vessels (208). As with other organ systems, the anatomic and clinical sequelae of viral infections within the lungs are related both to the infection and to the inflammatory response with which it is accompanied (225). Findings in early COVID-19 typically manifest as bilateral peripheral ground-glass opacities and consolidations, which may reflect diffuse alveolar damage when examined histologically (225). As with other viruses, organizing pneumonia can also be seen, as well as bronchiolitis, manifesting as bronchial wall thickening, centrilobular nodules, and tree-in-bud opacities (225). While early studies hypothesized a unique trajectory of lung compliance changes in COVID-19 ARDS (226), later studies suggest that COVID-19 ARDS is a heterogeneous entity composed of pathophysiologic phenotypes that may be managed similarly to previously described phenotypes of ARDS (227–231). The overlap with typical ARDS in severe COVID-19 may explain the benefit of dexamethasone in hospitalized patients with an increased supplemental oxygen requirement (232), similar to a recent trial studying dexamethasone in ARDS (233). Of note, the development of endotheliitis and microthrombi (208, 234) in COVID-19 does appear to be unique to SARS-CoV-2 as compared to other viral illnesses such as influenza H1N1 (208, 235), a finding which may reflect SARS-CoV-2 tropism for ACE2 on endothelial cells (197, 203, 205, 206) exacerbated by an inflammation-related hypercoagulable state (236). In line with such pathophysiology, the initiation of therapeutic-dose anticoagulation in non-critically ill patients has been shown to be effective in preventing the need for mechanical ventilation and improving survival to hospital discharge (237). Initiation of anticoagulation during critical illness, in contrast, has not been shown to improve survival, which may be due to the timing of microthrombi development and the momentum of the inflammation-related hypercoagulable state (238). Of note, overt pulmonary emboli (PE) have a pooled incidence of 21% in hospitalized patients with COVID-19 (239); treatment for PE in such scenarios involves standard anticoagulation.

#### 2.3.3 Acute cardiac dysfunction

Although the phenomenon itself is probably rare (240), numerous studies have revealed associations of COVID-19 to myocarditis (197, 240–243). One US hospital administrative database study among patients hospitalized from March 2020 to January 2021 showed that the risk for myocarditis was 0.146% for patients with COVID-19 compared to 0.009%, with an estimated 15.7 times increased risk for the syndrome (244). In several such cases, SARS-CoV-2 mRNA was shown to be present in the myocardium and was associated with an intense inflammatory response comprised of macrophages and CD8+ cytotoxic T-cells (245, 246). At least one patient exhibited no overt evidence of pulmonary SARS-CoV-2 infection (245). Other mechanisms of acute cardiac involvement may include stress-related (Takutsobu) cardiomyopathy (242) and myocardial infarction (240, 247) related to supply-and-demand mismatch (248) or overt coronary occlusion (249). COVID-19-associated IL-6 production can also affect cardiac function: IL-6 has been shown to exacerbate viral myocarditis (240), and a recently submitted study using cardiac organoids stimulated with IL-1β suggests cardiac dysfunction may be due to downregulation of sarcomere components, reduced sarcomere width, contraction amplitude, increased cardiac fibrosis, and prothrombotic vasculature, irrespective of direct infection with SARS-CoV-2 (250).

#### 2.3.4 Acute neurologic and psychiatric dysfunction

Neurological manifestations of COVID-19 appear to portend a worse outcome in terms of in-hospital mortality, hospital length of stay, and persistent functional disability (251). Neurological manifestations were estimated to occur in as many as 80% of patients hospitalized with COVID-19 (252), with the most common self-reported symptoms including headache (37%) and anosmia/ageusia (26%) and the most common syndromes including encephalopathy (49%), coma (17%) and stroke (6%) (252). Ischemic stroke appears to be more prevalent in SARS-CoV-2 infection (1.6%; 95% CI 1.1-2.3%) (253) than in influenza (0.2%; 95% CI, 0.0%-0.6%) (253, 254). COVID-19 patients with co-morbid pre-existing dementia are at exceptionally high risk for in-hospital delirium, itself associated with prolonged hospitalization, intensive care unit (ICU) admission, or in-hospital mortality (251). Other disorders include seizures, hypoxic/ischemic brain injury (251), encephalitis (255), critical illness neuropathy/myopathy, myalgias, and dizziness (251). Psychiatric disorders including psychosis (255), anxiety, depression and post-traumatic stress disorder (PTSD) were also reported in COVID-19 patients (256). Single-cell CSF analysis in patients with neurological manifestations demonstrated a dampened interferon response, an increase in de-differentiated monocyte populations, and exhausted CD4+ T-cell phenotypes (257). Other proposed mechanisms of neurological dysfunction include endothelial damage, local inflammation (258), direct effect on choroid plexus epithelium, glial cell tropism, retrograde transportation into the CNS from the olfactory bulb (256), and hypoxic-ischemic injury to the CNS (256).

#### 2.3.5 Immunologic effects on the kidney accompanying SARS-CoV-2 infection acute kidney injury

**Figure 4:**
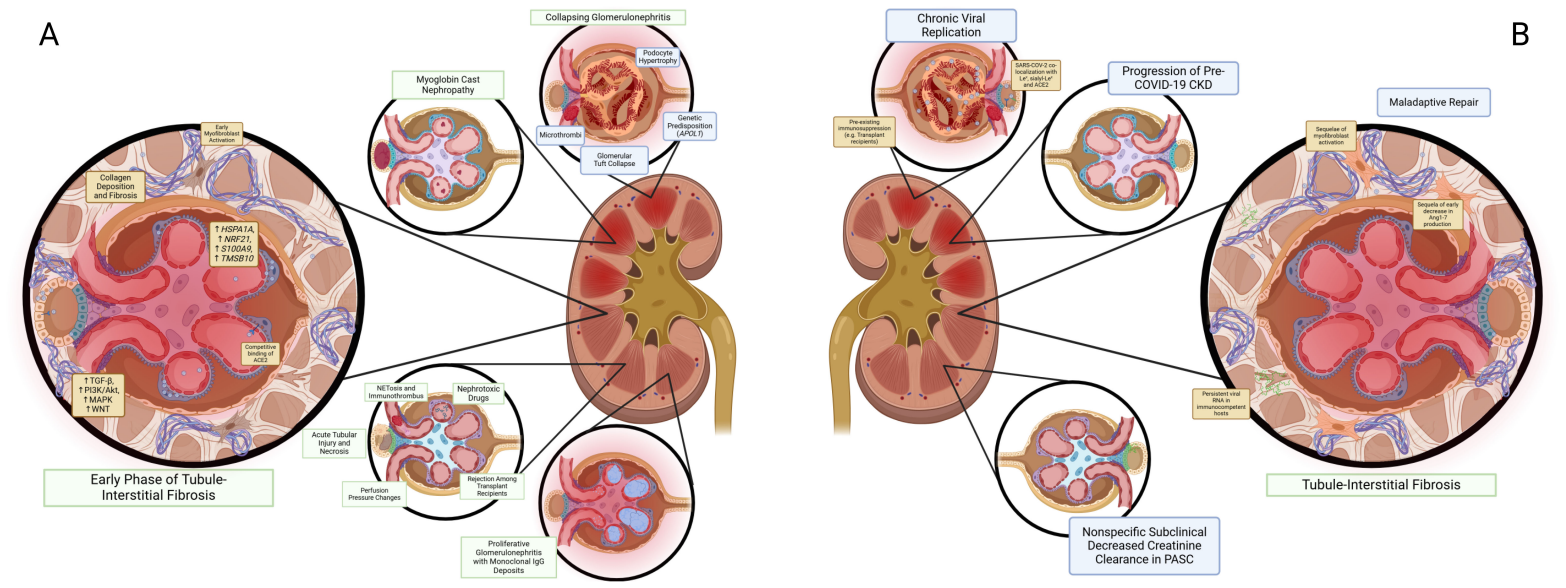
Inflammatory and immunologic consequences of SARS-CoV-2 in the kidney. **(A)** Histological and pathological mechanisms of acute kidney injury (AKI) in SARS-CoV-2 infection include collapsing glomerulonephritis, myoglobin cast nephropathy, proliferative glomerulonephritis with monoclonal IgG deposits, sequelae of nephrotoxic agents, acute tubular injury and necrosis (267), and perfusion pressure changes. More recent studies suggest that early tubule-interstitial fibrosis can occur in the setting of upregulated pro-fibrotic and pro-inflammatory mechanisms accompanied by myofibroblast activation, collagen, and ECM deposition. **(B)** Mechanisms of the development of chronic kidney disease (CKD) can include natural progression from AKI (414), which may occur as a result of maladaptive repair manifesting as early myofibroblast activation through proinflammatory pathways such as TGF-β, decreased Ang 1-7 production, and concomitant ongoing ECM and collagen deposition through mechanisms set off during the COVID-19 AKI phase. Direct damage to podocytes and proximal convoluted tubules via SARS-CoV-2 colocalization with Le^x^ and sialyl-Le^x^ (CD15s) in addition to ACE2 in the setting of chronic replication of SARS-CoV-2 may occur in immunocompromised hosts (365). Progression of pre-existing CKD may occur following COVID-19. In renal PASC, mildly decreased eGFR is likely to occur as a less clinically significant manifestation of SARS-CoV-2 infection. Additional mechanisms include CKD as a sequela of the array of etiologies of AKI in acute infection (414). Long-term sequelae of COVID-19 may also include subclinical decreased creatinine clearance (366), presumably through the mechanisms described for more overt CKD phenotypes. Persistent viral RNA may be present in immunocompetent patients and has been suggested as one mechanism for PASC (291).

The kidney is unique in its high concentration of ACE2, its increased vascularity, and its exposure to systemic pathogens (16). ACE2 is co-expressed with TMPRSS2 in podocytes and tubule epithelial cells as it is in the lungs (259). Furin and CD147, which bind SARS-CoV-2, are likewise expressed in the kidney (260). Immune cells in the healthy human kidney are sparse and are primarily CD4+ and CD8+ T cells, with a smaller percentage of NK cells, B cells (17), and CD14+, CD16+ and CD68+ myeloid cells (18). Infection with SARS-CoV-2, a shift occurs from an upregulation in proinflammatory genes such as HSPA1A in podocytes and JUN1 in mesenchymal clusters (19) can accompany selective immune suppression of lymphocytes mediated through T-cell immunoglobulin and mucin-domain containing-3 (TIM-3) and Programmed cell death protein 1 (PD-1) (20). A 2022 review suggests that resident immune cells may mediate inflammation by TNFα release, IL-34-mediated necrosis, and NLRP3 inflammasome production (266). ICAM-1, E- and P-selectin upregulation may be associated with neutrophil and circulating immune cell recruitment, platelet activation, NETosis (266), and immunothrombosis. While tropism for the kidney had previously been debated (255), the detection of SARS-CoV-2 RNA in a case series of kidney autopsies (264) suggests a role for direct infection. One elegant study built on the finding of increased extracellular matrix (ECM) in pathological specimens to explore an association with tubule-interstitial nephrosis (260). In this study, single-cell RNA sequencing of SARS-CoV-2-infected human-induced pluripotent stem-cell-derived kidney organoids confirmed the presence of SARS-CoV-2 in podocytes, proximal tubular cells, and myofibroblasts. Increased collagen one protein expression was seen in SARS-CoV-2 infected organoids compared to controls, a finding abrogated with a TGF-β blocker (SB431542). Moreover, the upregulation of HSPA1A, NRF21, S100A9, and TMSB10 proinflammatory genes was seen in podocytes, as was CCN1, JUN, and NFKBIA in mesenchymal clusters. Pathways upregulated among SARS-CoV-2-infected cells included TGF-β, PI3K/Akt, MAPK, and WNT signaling in proximal tubular cells and mesenchymal clusters (260). Upregulated IL-6 and STAT3 signaling may be important in COVAN-mediated disease (265). These results support a hypothesis that SARS-CoV-2 promotes tubule interstitial fibrosis through profibrotic pathways, at least in severe disease (**Figure 4**).

Of note, patients with ESRD may already exhibit exhausted T-cell phenotypes (259). As discussed above, infection with SARS-CoV-2 is likewise associated with lymphopenia and exhausted T-cell phenotypes (260), which may indicate exacerbated immune dysregulation in such patients. Patients with ESRD have been shown to produce durable antibody anti-nucleocapsid and anti-RBD antibodies at least 6 months post-infection (261, 262), although this was not found to correlate with a decreased likelihood of reinfection in patients undergoing hemodialysis (263). While not the subject of this review, a more recent study noted that patients with both mRNA-1273 vaccination as well as prior infection with SARS-CoV-2 exhibited more robust neutralizing antibody production and S-specific B-cells were higher among patients with chronic kidney disease, and that the percentage of S-specific memory CD4+ and CD8+ T-cells were higher among patients who were dialysis-dependent than healthy hybrid controls (264).

Early studies in China suggested an incidence of AKI in 4.7% of all patients presenting with SARS-CoV-2 infection by Kidney Disease: Improving Global Outcomes (KDIGO) Criteria (265, 266). In another study, AKI was reported in approximately 25% of hospitalized patients with COVID-19 (267). The International Severe Acute Respiratory and Emerging Infections Consortium (ISARIC) World Health Organization (WHO) Clinical Characterisation Protocol UK (CCP-UK) for Severe Emerging Infections was among the largest epidemiological studies performed during the COVID-19 pandemic (268). Among the 85687 patients in this multicenter cohort, 2198 (2.6%) ultimately underwent acute renal replacement therapy (RRT) (long-term dialysis patients were excluded.) Among 41294 patients with available serum creatinine, 13000 (31.5%) had biochemical AKI: 8562 had stage 1 (65.9%), 2609 stage 2 (20.1%) and 1829 stage 3 (14.1%), with a concurrent increased risk in 28-day mortality by AKI severity (stage 1: aOR 1.58 (1.49–1.67); stage 2: aOR 2.41 (2.20–2.64); stage 3 aOR 3.50 (3.14–3.91); and RRT aOR 3.06 (2.75–3.39) (268). As summarized in the 2022 study, predominant risk factors for RRT were chronic kidney disease (CKD) (aOR 3.41; 95% CI=3.06–3.81), male sex (aOR 2.43; CI=2.18–2.71) and Black race (aOR 2.17; CI=1.79–2.63). Primary risk factors for biochemical AKI were admission respiratory rate >30 breaths per minute (aOR 1.68; CI=1.56–1.81), CKD (aOR 1.66; CI=1.57–1.76), and Black race (aOR 1.44; CI=1.28–1.61) (268).

In addition to the acute effect of COVID-19 and its consequences on the kidney, an early phenome-wide association study (PheWAS) of patients showed a significant increase in the likelihood of hospitalization among those patients with stage 4 CKD and above (Stage 4 CKD: OR 2.90, 95% CI: 1.47, 5.74), stage 5 CKD or dialysis (OR 8.83, 95% CI: 2.76, 28.27) (269). The odds of hospitalization were especially high among kidney transplant recipients (OR 14.98, 95% CI: 2.77, 80.8) (269), with plausible mechanisms including transplant rejection as seen in a 2021 biopsy series (270).

In the largest biopsy series among patients with SARS-CoV-2 infection (45.4% with AKI and 42.6% for proteinuria with or without AKI), among whom 44.6% were African American, the most common diagnosis was collapsing glomerulopathy (25.8%) (270). Of those patients, 91.7% had high-risk *APOL1* genotypes (270) [G1/G1, G1/G2, or G2/G2 (271)]. Increased rates of myoglobin cast nephropathy (3.3%), proliferative glomerulonephritis with monoclonal IgG deposits (PMIG) (1.7%), and rejection among transplant patients (61.4% of transplant patients) were also seen when compared to historical controls (**Figure 4A**) (270). Lower rates of arterionephrosclerosis, diabetic nephropathy, and IgA nephropathy were also noted within this cohort (270). Only 3.7% of biopsy specimens within this series exhibited SARS-CoV-2 N protein by immunohistochemistry, none of which were positive by *in situ* hybridization (272).

The unique association of COVID-19 and collapsing glomerulopathy has led to the identification of an entity known as COVID-19 associated nephropathy (COVAN), which is thought to be similar to HIV-associated nephropathy (HIVAN) (273). Mechanisms of COVID-19-induced collapsing glomerulopathy—a variant of focal segmental glomerulosclerosis (FSGS) characterized by collapse of the glomerular capillaries with hypertrophy of the overlying podocytes (271)—may include direct infection as well as thrombotic microangiopathy or other ischemic insults (271). In addition to the above-mentioned immunologic processes, mechanisms of COVID-19-related AKI include acute tubular injury (ATI) and acute tubular necrosis (ATN) from various etiologies, including systemic hemodynamic instability (267). Proteinuria can occur in COVID-19-related AKI and is typically low molecular weight, indicative of tubular injury (267). Other proposed mechanisms include intravascular hypovolemia, increased right heart filling pressures secondary to pulmonary pathology with associated renal venous vascular congestion, rhabdomyolysis, glomerulonephritis, and collapsing glomerulopathy (267). Additional patho-etiologies include endothelial inflammation, microthrombi formation, and thrombotic microangiopathy (267). NET-related immunothrombosis may be implicated, particularly in patients with proteinuria (111). Nephrotoxic agents administered during the course of hospitalization may also cause AKI through varied mechanisms (267) (**Figure 4**).

#### 2.3.6 Gastrointestinal damage and dysfunction

Up to 50% of patients with COVID-19 develop GI symptoms such as nausea, abdominal pain, and diarrhea (274). SARS-CoV-2 can be detected in the stool, suggesting a role for fecal-oral transmission (275). Enterocytes can be directly infected with SARS-CoV-2 (276). Inflammatory infiltration of the intestine comprised of neutrophils, macrophages, and lymphocytes may be seen in severe COVID-19 (276). Studies in human small intestinal organoids reveal a response similar to that seen in the lungs (277), with low levels of interferons I and III and high levels of chemokines such as IP-10 and CXCL10 (278) skewing the immune response towards inflammation with less potent antiviral activity (274, 277). Systemic inflammation, including cytokine release, can induce leakiness in the gut mucosal barrier, leading to exposure to LPS and β-D-glucan and the development of neutrophil extracellular traps (NETs) (279). Alterations in the gut microbiome in SARS-CoV-2 infection are thought to contribute to COVID-19-mediate inflammation, including through increased gut permeability and associated endotoxemia (280). In one study of metagenomic and metaproteomic profiles of COVID-19 patients, an increase in opportunistic pathogenic species such as Burkholderia contaminans was seen along with a decrease in commensal bacteria and was associated with illness severity, highlighting the role of the microbiome (281). Other studies showed increases in Escherichia coli, Klebsiella pneumoniae and Enterococcus faecalis (282). Antibiotic use during the pandemic is also likely to have contributed to microbial dysbiosis (282). The reduction in short-chain fatty acids, metabolites of a normal microbiome that can serve as fuel for enterocytes and regulators of innate and adaptive immunity, may help to explain COVID-19-related microbial dysbiosis (282).

## 3 Post-acute and chronic immunologic and inflammatory response to SARS-CoV-2

Early waves of the pandemic were followed by the growth of a population of patients who continued to suffer from physical and neuropsychiatric symptoms (283) as well as overt organ damage (284). In the UK’s National Health System (NHS), for instance, approximately one-third of patients who were hospitalized with COVID-19 were readmitted to the hospital within 5 months (284), implying a substantial individual and public health burden. A collection of symptoms, including shortness of breath, fatigue, memory loss, GI distress, and anosmia, has come to be called “long COVID” or post-acute sequelae of COVID-19 (PASC) (285). In a recent meta-analysis that alluded to substantial study heterogeneity (I^2^ = 100%, P < 0.001), global pooled prevalence of any post-COVID-19 condition is estimated to be approximately 43% (95% CI, 39-46%) (283), with fatigue being the most common symptom reported (23%; 95% CI, 17-30%) and with symptoms more likely to be present in patients who were hospitalized with COVID-19 (283). Other studies reported at least one PASC symptom in half of all survivors of COVID-19, extending to at least 6 months following hospitalization (median 54.0% (IQR 31.0%-67.0%) (286). Pre-existing comorbidities likely predispose patients to PASC: A recent study showed a prevalence of 2.8% to 5.5% in people with pre-existing health conditions as compared with 1.8% in healthy controls (287). Mechanisms of post-COVID organ dysfunction and PASC are an area of current and active interest (285, 288) with definitions of PASC being in a state of flux likely accounting for the heterogeneous findings (283).

### 3.1 Systemic inflammatory response to COVID-19: long-term effects from acute response and long-term immune response

#### 3.1.1 Persistent viral RNA

Pathological studies have shown that SARS-CoV-2 viral RNA can persist and replicate throughout the body in multiple organs, including the heart, lung, brain, small intestine, and adrenal gland, for at least seven months post-infection (289). Circulating spike protein has been detected in approximately 60% of patients diagnosed with PASC for up to 12 months post-infection compared to those not diagnosed with PASC (290). While the clinical implications are unclear (289), the above findings and several other studies have fueled the hypothesis that PASC may be related to viral persistence (291). The presence of antibodies to spike antigen in 60% of COVID-19 survivors with PASC, in contrast to 0% of such antibodies in patients without PASC, indirectly points to a viral reservoir promoting persistent inflammation (290, 292). Of note, although less common than with DNA viruses, the phenomenon of persistent RNA virus is not unique to SARS-CoV-2 (293, 294) and has been seen with adenovirus, enterovirus, parvovirus B19 (293, 294), rhinovirus, respiratory syncytial virus (RSV) and others, and correlated with cardiomyopathy, asthma, and chronic pulmonary disease (293). The attention to virus-associated clinicopathology that the SARS-CoV-2 pandemic has spurred may thus prove impactful for many chronic illnesses.

#### 3.1.2 Altered immune activation with co-presentation of EBV

Among the mechanisms studied for persistent symptoms in patients who have recovered from acute COVID-19 is altered immune activation to co-persistent Epstein-Barr Virus (EBV) (291). A 2021 study found that those patients who suffered from PASC symptoms were more likely to have evidence of EBV reactivation manifesting as early antigen diffuse (EA-D) IgG or viral capsid IgM (295). A later study showed that detection of EBV DNA was more than twice as common (27.1% vs. 12.5%) in patients diagnosed with COVID-19 (296). In another study, EBV viremia was seen in 14% of patients diagnosed with acute COVID-19 (285). In that same study, EBV viremia was associated with specific symptoms of PASC such as fatigue (285). Given the association of EBV with a number of clinical conditions ranging from chronic fatigue syndrome and multiple sclerosis (297), it’s role in acute and chronic forms of COVID-19 should not be ignored. Of note, re-activation of dormant infections is likely not unique to EBV in COVID-19. Similar phenomena have been suggested with tuberculosis (298) and toxoplasmosis (299), and may yet be the subject of further study.

#### 3.1.3 Persistent cytokine release and the role of inflammasomes

Cytokines such as IL-2, IL-6, IL-17, TNFα, and IFN-γ may be persistently elevated for weeks during active acute COVID-19 (139) and may continue to be elevated in the early post-acute phase of COVID-19 (300, 301). In particular, IL-6, IFN-β, IFN-γ, and IFN-λ2/3 are associated with symptoms of PASC at least eight months after acute infection (301). Macrophages and monocytes may be responsible for the ongoing release of IL-1β, IL-6, and TNF (302). Hypotheses about the relevance of persistently elevated cytokines in PASC include low-grade chronic peripheral inflammation leading to microglia dysfunction and neuro-inflammation, which may explain “brain fog” and similar symptoms. This mechanism has yet to be proven (303). Among the mechanisms of cytokine release, the inflammasome-mediated pyroptosis pathway has been implicated in COVID-19 (67) and may involve NLRP3 (304). NLRP3 is associated with the development of such autoimmune conditions as rheumatoid arthritis (RA), systemic lupus erythematosus (SLE), systemic sclerosis (SSc), and inflammatory bowel disease (IBD) (305). Therefore, persistent NLRP3-mediated inflammation via IL-1β may be at play in patients with PASC (306). In a study among PASC patients with lung fibrosis, activation of the Absent in Melanoma 2 (AIM2) inflammasome was associated with the release of IL-1α, IFN-α and TGF-β, a finding not seen in non-PASC patients (306). Of note, in that same study, the provocation of NLRP3 with LPS or ATP did not induce IL-1α release (306). The significance of inflammasomes in acute and chronic forms of COVID-19 remains the subject of ongoing study (307).

#### 3.1.4 Autoantibodies

Autoantibody development may be associated with the emergence of PASC (285). Several patients can develop autoantibodies early in the course of illness (308), as well as convalescent patients (285) (**Figure 2E**). Among a cohort of patients with life-threatening COVID-19, 10.2% were found to have IgG autoantibodies to type I IFNs IFN-α2 and IFN-ω (309). A unique sequencing technique called Molecular Indexing of Proteins by Self-Assembly (MIPSA) further identified type III-anti-IFN-λ3 autoantibodies in patients with severe COVID-19 (415). Another study revealed that one out of every four hospitalized patients with COVID-19 in their cohort had anti-nuclear antibodies (ANA) of various titers and patterns (308). Following acute infection, autoantibodies can linger or develop anew. In a recent study, 44% of patients with symptoms of PASC were found to have autoantibodies, including the ANAs Jo-1, Ro/SS-A, La/SS-B, U1-snRNP, P1, and anti-IFN-α2, approximately 2-3 months after initial symptom onset (285). Intriguingly, immune profiles of patients with PASC may be similar to those with SLE (285, 310). While it is difficult to ignore the possibility of pre-existing autoantibodies in certain patients before the development of COVID-19, a 2022 study points out that few of the patients found to have autoantibodies early in the course of illness had clinical evidence of autoimmune disease before COVID-19, suggesting a pre-existing subclinical autoimmune process that COVID-19 exacerbates (285).

#### 3.1.5 T-cell and B-cell subpopulations

In the early post-recovery phase, lymphopenia may persist (300). Following acute infection, CD4+ and CD8+ T-cell populations that upregulate genes associated with inflammatory regulation are preferentially expanded, and those associated with effector functions are contracted (285). In some patients, however—particularly those experiencing GI symptoms of PASC—populations of cytotoxic CD4+ and CD8+ T-cells continue to evolve into new clones, suggesting a role for T-cell-mediated inflammation in PASC (285). Additional studies have shown that naive CD127^low^GzmB^−^CCR7^+^CD45RA^+^CD27^+^CD8^+^ T-cells, naive CD127^low^TIM-3^−^CCR7^+^CD45 RA^+^CD27^+^CD4^+^ T-cells, and naive CD127^low^TIM-3^−^CD38^low^CD27^−^IgD^+^ B cells are absent eight months post-infection in patients with PASC, suggesting ongoing activation of normally naïve T-cell and B-cell subsets (301). As noted above, T_H_17 cells may be preferentially expressed in the hyperinflammatory environment in COVID-19, which includes TGF-β and IL-6 (153, 311). T_H_17 cells have been associated with several autoimmune diseases, including asthma, multiple sclerosis, SLE, and RA (153). Specific cell signatures in T-cells, B-cells, and NK cells have defined different immune endophenotypes among patients with PASC (285). The interplay between T-cells and other viruses in a patient’s virome (312), such as EBV and CMV, may also affect the development of PASC symptoms (285). Additional research can help further describe clinical and endophenotypes following infection with SARS-CoV-2 and elucidate the significance of these findings.

### 3.2 Post-acute and delayed inflammation-related end-organ injury following SARS-CoV-2 infection

#### 3.2.1 Multisystem inflammatory syndrome in children and adults (MIS-C; MIS-A)

Multisystem inflammatory syndrome in children (MIS-C) and adults (MIS-A) are distinct acute hyperinflammatory illnesses involving end-organ dysfunction (313) that can occur 2-12 weeks after acute infection with SARS-CoV-2 (314). Diagnostic criteria for MIS-C vary but typically include age <21 years, persistent fever, involvement of ≥2 organ systems [e.g., cardiac dysfunction (seen in 86.5% in one study), dermatologic or mucocutaneous involvement (70.9%), and GI involvement (90.9%) (315)] and inflammatory markers in the context of a preceding SARS-CoV-2 infection (313). Diagnostic criteria for MIS-A presently include severe cardiac illness, dermatologic and conjunctival manifestations, and elevated inflammatory markers in the context of recently positive SARS-CoV-2 PCR, antigen, or antibody testing in patients >21 (316). It is important to note that these two syndromes, while similar in name, likely reflect distinct pathological processes. MIS-C is more common than MIS-A and exhibits strong similarities to Kawasaki Disease (313). Both MIS-C and MIS-A exhibit similarities to macrophage activation syndrome (MAS), secondary hemophagocytic lymphohistiocytosis (HLH), and toxic shock syndrome (317) and may be equivalent to an immunologic aftershock following a sometimes-benign initial infection (317). MIS-C may be distinguishable from severe COVID-19 by cytokine profile differences such as increased IL-10 in MIS-C (317). Coronary artery aneurysms have been reported in MIS-C (318); both MIS-C and MIS-A can be associated with myocarditis (319–321), which can progress to cardiogenic shock (321) and may respond to immunosuppressive agents (13, 320, 321).

### 3.3 Chronic end-organ dysfunction following infection with SARS-CoV-2

The early effects of SARS-CoV-2 infection and the associated immune response can have a long-term impact on multiple organ systems. Post-acute sequelae of COVID-19 (PASC), also called Long COVID, long-haul COVID, and other names, is now widely recognized as a clinical entity carrying its own *International Classification of Diseases, Tenth Revision, Clinical Modification* (ICD-10-CM) diagnosis (U09.9 Post COVID-19 condition, unspecified) (322). The convenience of a unifying diagnostic code belies the sundry manifestations of this condition. Patients who have recovered from COVID-19 may display clinical and pathological evidence of end-organ dysfunction, which may overlap those underlying PASC (**Figure 3B**). Pre-existing factors such as older age, female gender, higher BMI, and previous hospitalization are associated with the development of symptoms of PASC (323).

#### 3.3.1 Cardiac dysfunction

A large US Veterans’ study found that the risk of cardiovascular disease, including inflammatory heart disease, was increased beyond 30 days after acute SARS-CoV-2 infection, even when mild (324). COVID-19 in the acute phase (defined as ≤ 21 days) and in the post-acute phase (> 21 days) were shown to be associated with an increased risk of cardiovascular disease than contemporary controls [hazard ratio (HR) for acute phase: 4.3 (CI=2.6-6.9); HR for post-acute phase: 1.4 (CI=1.2-1.8)], findings which also held for historical controls whose population health was not overtly impacted by COVID-19 as a whole (325, 326). The risk of ischemic heart disease, heart failure, myocardial infarction, MACE, myocarditis, and pericarditis were all increased in the 12 months following acute SARS-CoV-2 infection (324). The risks of atrial fibrillation, ventricular arrhythmias, and other dysrhythmias were also higher (324). Myocarditis carried the highest risk (HR 5.38; CI: 3.80-7.59) of all such outcomes, which remained increased for COVID-19 patients even when vaccination was accounted for (324). A retrospective cohort study from a US health plan reported similarly increased risks in several of the outcomes mentioned above, as well as an increase in the diagnosis of hypertension (327). The risk of cerebrovascular disorders like stroke and TIA was also increased (324). While the impact of pre-existing comorbidities should not be ignored (287), subgroup analyses of the 2022 Veteran study suggested that COVID-19 was itself a risk factor for future cardiovascular disease *regardless of pre-COVID-19 risk* (324, 328). Moreover, COVID-19 disrupted the care of patients with cardiovascular disease and was associated with an overall increase in mortality due to these conditions (326).

#### 3.3.2 Pulmonary dysfunction

Pulmonary hypertension (and associated right-sided cardiac dysfunction) (329) can be observed as a result of pulmonary fibrosis (330) and prior pulmonary emboli (i.e., Group 3 and Group 4 pulmonary hypertension, respectively) (331). Patients with pulmonary emboli may exhibit functional limitations and dyspnea (332) even in the absence of overt pulmonary hypertension. Notably, the risk of new-onset PE [as defined by hazard ratio (HR) (324)] and a new diagnosis of pulmonary hypertension (327) have also been found to be higher in patients who have had COVID-19. On imaging, early infection with SARS-CoV-2 can give way to air trapping in patients with PASC seen in a post-COVID-19 clinic 42-204 days after diagnosis (333). This CT finding suggests persistent small airway disease (333), presumably reflective of post-viral constrictive bronchiolitis, and is typically not associated with large airway obstruction on pulmonary function tests (PFTs) (333, 334). Patients with more severe infections (i.e., those requiring an ICU stay) were additionally found to have GGOs, architectural distortion, honeycombing, scar, or traction bronchiectasis accompanied by restrictive lung physiology and a reduction in DLCO (333, 335). Pulmonary fibrosis and interstitial lung disease (ILD) following COVID-19 (336–338) may be due to an initial hyperproliferation of pathologic fibroblasts acting via TGF-β signaling (339). Post-COVID pulmonary complications are likely to be related to disease severity, as suggested by an association with the inflammatory markers ESR and CRP (340), as well as to pre-existing comorbidities (287). Observational studies are now underway to elucidate the mechanisms and clinical trajectory of post-COVID-19 ILD (336). The risk of a new diagnosis of sleep apnea appears to be higher following hospitalization for COVID-19 (327).

#### 3.3.3 Neuropsychiatric symptoms

Neuropsychiatric symptoms can be a prominent component of PASC and are typically characterized by fatigue, cognitive dysfunction, and post-exertional malaise (341). A recent survey study confirmed similarities between fibromyalgia and chronic fatigue syndrome with respect to self-reported fatigue, cognitive function, anxiety, depression, kinesiophobia, pain, and physical dysfunction, although patients with PASC had lower levels of fatigue and pain (342). Neurocognitive testing confirmed impairments in 46% of this preselected group of patients (343) and has been reported in other populations (344). Persistent neuroinflammation and endothelial injury are among the proposed mechanisms for this phenomenon (345).

Laboratory abnormalities among patients with PASC include reduced cortisol levels (346) and a reduced circulating serotonin level (347). A compensatory increase in ACTH was not seen among patients with low cortisol, which may reflect a blunted stress response in patients with PASC (346). The correlation between viral-RNA-induced type I interferons and reduced serotonin is thought to be mediated by reduced tryptophan uptake and storage and hypercoagulability, as shown in a recent elegant study from 2023 (347). In that same study—which opened the door to a compelling mechanism underlying PASC—cognitive dysfunction correlated with peripheral serotonin depletion (347).

In post-hospital discharge patients with symptoms of PASC, the most common MRI finding was scattered white matter lesions (343). An increased risk of incident ischemic and hemorrhagic stroke, encephalitis, encephalopathy, migraine, seizures, sensory disorders, peripheral nervous system disorders, musculoskeletal disorders, Guillain-Barre Syndrome, and extrapyramidal disorders was seen among patients followed for 12 months following acute SARS-CoV-2 infection, with an estimated HR for any neurologic sequela of 1.42 (1.38-1.47) (344). In one comprehensive NIH study of 12 patients with neurologic sequelae of SARS-CoV-2 infection, mild cognitive impairment (Montreal Cognitive Assessment Score (MoCA) < 26) was seen in half of the patients, with deficits predominantly seen in short-term memory (348). No MRI abnormalities were seen in those patients. Immunophenotyping of CSF revealed higher frequencies of antibody-secreting B cells and PD-L1-expressing monocytes and lower frequencies of CD4+ and CD8+ effector memory cells (348). It should be noted that one-third of patients included in the study had a prior history of resolved long-term disability due to a preceding infection (amoebiasis due to *Entamoeba histolytica*, infectious mononucleosis, Lyme disease, and severe sepsis due to group A Streptococcus) (348).

#### 3.3.4 Persistent renal dysfunction and chronic kidney disease

In a US health plan cohort, renal dysfunction, as defined by a composite of AKI and CKD, was found to be more likely after a diagnosis of COVID-19 in hospitalized patients with a pre-existing condition (327). Other studies reported an increased likelihood of a new diagnosis of CKD within the first 4-6 months following COVID-19 in addition to AKI (284, 349). A large VA health system study also revealed an increase in the diagnosis of CKD following COVID-19 infection in hospitalized and intensive care unit patients (HR, hospitalized patients=1.36 (CI=1.24-1.49), and HR for ICU patients=1.88 (1.66-2.13) (350).

CKD is known to be associated with altered immunity. ESRD has likewise been defined in part as a state of “acquired immunodeficiency” by Vanholder and Ringoir in 1993 (351), although simultaneously heightened levels of inflammation and immune activation have also been described noted (352). In ESRD patients, CD14+CD16+ monocyte populations are expanded, cytokine production and chemokine expression is increased, as is basal ROS production (352, 353). LDL likewise elicits a stronger pro-inflammatory response (354). In contrast, a decreased number of DCs with impaired function (355) are seen, circulating PMNs have decreased phagocytic capacity, naïve and central memory T cells are depleted (with a reduced CD4+/CD8+ T cell ratio), CD4+CD25bright+FoxP3+ Treg populations are both diminished and impaired, and B cell populations are likewise diminished (352).

An early PheWAS study cited above pointed to a strong association between pre-existing Stage 4 CKD, Stage 5 CKD, and dialysis with the likelihood of hospitalization with even higher odds among kidney transplant recipients ￼, suggesting a disproportionate impact among those with pre-existing renal disease as well as the bidirectional interplay between COVID-19 and CKD (356). Among 758 patients in the Health Outcome Predictive Evaluation of COVID-19 (HOPE COVID-19) Study, an eGFR of < 60 ml/min/1.73m^2^ was associated with a higher risk of in-hospital mortality (eGFR > 60: 18.4%; eGFR 30-60: 56.5%; and eGFR < 30: 65.5%; p < 0.001), multi-organ failure, and sepsis (356, 357). Notably, while only 8.5% of the HOPE-COVID-19 cohort had documented CKD on admission, 30.6% (N=322) presented with eGFR < 60 ml/min/1.73m^2^, suggesting a role in the development of AKI as described above (356, 357).

Mechanisms of the development of CKD in the general population include the progression of changes set in motion through COVID-19 AKI, particularly as the development of AKI itself can be associated with the eventual development of CKD (358). Acknowledging the unique etiological mechanisms of the initial AKI insult (359, 360), injury of proximal tubule cells in severe AKI may result in cell cycle arrest in the G2/M phase, which leads to the secretion of TGF-β and connective tissue growth factor, both of which mediate fibrosis through c-jun NH2-terminal kinase (JNK) signaling (359, 361). Following AKI, animal models have shown that aberrant activation of developmental pathways such as Hedgehog and Wnt/β-catenin can also promote fibrosis (359). Persistent mitochondrial dysfunction following the initial insult may also play a role in developing fibrosis and persistent inflammation (359). Resident *P0-Cre* fibroblasts in the renal cortex and medulla (359) may transdifferentiate into αSMA-positive myofibroblasts and promote fibrosis (359); *Gli1-Cre* fibroblasts are likewise related to end-organ fibrosis and may be therapeutically targeted (362).

A 2022 study reviewed a biopsy series and confirmed the presence of tubule interstitial fibrosis in patients with COVID-19 as compared to age, sex, and comorbidity-matched controls ￼; in an elegant study described above for AKI, infection of human iPSC-derived organoids elucidated SARS-CoV-2-specific pathways of tubule-interstitial fibrosis mediated through direct infection (19). Early pro-inflammatory, fibroblast activation, and myofibroblast differentiation, including those mediated by TGF-β, NFκB, and JAK-STAT, were upregulated in podocytes, proximal tubule cells (PTC), and fibroblasts, with a concomitant increase in ECM and collagen deposition (19). Additional mechanisms are described above. Further, the initial binding of SARS-CoV-2 with ACE2 in the kidney in acute infection limits its bioavailability for Ang1-7 production, leading to fibrosis (363) and maladaptive repair (359, 364). Mechanisms in immunocompromised patients may involve direct damage to podocytes and proximal convoluted tubules via SARS-CoV-2 colocalization with Le^x^ and sialyl-Le^x^ (CD15s) in addition to ACE2 in the setting of chronic replication of SARS-CoV-2 as seen on biopsy from a patient with splenic marginal cell lymphoma (365).

Much of the research on COVID-19 AKI and CKD has been performed among patients with more severe illness. A 2022 study of a primarily ambulatory cohort of patients in Hamburg, Germany, showed a decreased eGFR (regression estimate -2.35mL/min/1.73m^2^ (CI= -4.28, -0.42); Bonferroni adjusted p=0.019) as compared to matched controls (366). Mechanisms or histological specimens were not described in this study. However, it is conceivable that SARS-CoV-2-mediated AKI and subsequent CKD exist on a spectrum, and likely that if so, the above mechanisms are at play in what may be considered “renal PASC.” As noted above, persistent viral RNA may be among the mechanisms in renal PASC (291).

#### 3.3.5 Other organ systems

In a large cohort of post-discharge patients in the UK’s NHS, the rate of new diagnoses of chronic liver disease increased following hospitalization for COVID-19 (284). Diagnosis of diabetes was also increased (284). A large VA cohort identified an excess burden of diabetes beyond thirty days after COVID-19 diagnosis in non-hospitalized patients, in addition to lipid disorders and obesity (350). An increased burden of esophageal disorders, gastrointestinal disorders, and dysphagia was also seen, as was incident elevated alanine aminotransferase (ALT) levels (350). As described above in the context of acute infection, mechanisms of excess inflammation in post-infectious symptoms include alterations in the gut microbiome, with acute and post-infectious inflammation possibly reflecting perturbations in the gastrointestinal mucosal barrier (367). An extra burden of skin disorders, arthralgias, and arthritis was also seen in the VA cohort (350). Thyrotoxicosis related to both subacute thyroiditis and Graves’ Disease has been reported (368). Reports of hormonal changes in male and female patients vary; an increase in menstrual cycle has been reported (368, 369). Orchitis and epidydimo-orchitis may occur in acute COVID-19 (368). The immunologic changes that accompany SARS-CoV-2 may also prove to be associated with long-term pathophysiology in bone (370). Finally, vasculitis (371), myositis (371, 372), and rhabdomyolysis (371) have been reported in acute COVID-19; arthritis (350), including inflammatory arthritis (371), has been reported in the post-infectious period. A comprehensive summary of the end-organ effects on various organ systems affected in both acute and post-infectious COVID-19 is shown in **Table 3** and **Figure 3**.

**Table 3:** Acute and post-acute end-organ dysfunction in COVID-19.

Organ system	Acute effects	Post-acute effects
Cardiovascular	Myocarditis (197, 240–243); stress cardiomyopathy (242); myocardial infarction (240, 247–249); pericardial effusion, tamponade (371)	Shortness of breath (285); coronary artery aneurysms (MIS-C) (318); myocarditis [in the absence of (324) or presence of MIS-C or MIS-A (319–321)]; risk of ischemic heart disease, MI, MACE, heart failure, atrial fibrillation, ventricular and other arrhythmias may be increased beyond acute infection (324); increased likelihood of diagnosis of hypertension (327)
Respiratory	Hypoxemic respiratory failure (222); pneumonia (219, 224); ARDS (224); PE (239)	Shortness of breath (285); pulmonary fibrosis (330, 336); ILD (336), pulmonary hypertension (327, 329); PE (324); diagnosis of sleep apnea (327)
Renal	AKI (267, 327); proteinuria, tubular injury (267); hematuria (371); need for RRT (367); hyponatremia (368)	CKD (327), decreased eGFR, ESRD (310)
Nervous/Psychiatric	Headache, anosmia/ageusia, encephalopathy, coma, stroke (252); encephalitis (255); delirium (251); ischemic stroke (253, 254); anxiety, depression (251)	Memory loss, fatigue (285, 341), neurocognitive impairment (341, 344), encephalopathy, encephalitis (344), migraine headache (344), anosmia (285), post-exertional malaise (341), risk of TIA, risk of stroke (324) [both ischemic and hemorrhagic (344)], seizures (344), extrapyramidal disorders (344), peripheral nervous system disorders (344), Guillan-Barre Syndrome (344); dysautonomia, postural tachycardia syndrome (POTS) (310)
Enteral	Nausea, abdominal pain, diarrhea (274); perturbation in GI mucosal barrier may be implicated in both acute COVID-19 and PASC (310)	Diagnosis of esophageal disorders, dysphagia, gastrointestinal disorders (350), chronic liver disease (284); elevated ALT (350); potential for alteration of gut microbiome (367)
Hematologic	Lymphopenia (408); neutrophilia, prolonged PT 34783405} and aPTT (409), decreased fibrinogen, elevated D-dimer (408), hypercoagulability (409), VTE (239), thrombocytopenia, disseminated intravascular coagulation (DIC), cytokine abnormalities (409, 410); hyperferritinemia (410)	Increase in amyloid deposits in plasma (411), VTE/PE (324, 412);
Endocrine	Pituitary apoplexy (particularly in patients with pre-existing macroadenoma) (368); subacute thyroiditis- and Graves’-associated thyrotoxicosis (368); low TSH and low T3 (368), adrenal insufficiency, adrenal hemorrhage, adrenal infarction (368); alterations in serum cortisol, with higher levels associated with more severe disease (368); new onset type 1 diabetes mellitus (368); decreased menstrual volume, prolonged menstrual cycle (33288478; 34543404}; varying reports of E2, progresterone, AMH, and testosterone levels in female sex (368); epidydimo-orchitis, hypogonadism (possibly physiologic response to stressor) (368)	Persistent hyperglycemia (368); diagnosis of diabetes (284, 350, 368); diagnosis of lipid disorders and obesity (350); possible effect on bone density (370); cortisol downregulation may be associated with PASC (285, 288)
Rheumatologic	Production of pro-inflammatory cytokines (410); arthralgia, myalgia (371); myositis (371, 372); rhabdomyolysis (371); vasculitis (small, medium and large vessel) (371)	Arthralgias, arthritis (350) (including inflammatory arthritis (371); autoantibodies to IFN-I, ANA (Ro, La, Jo-1, P1, and U1-snRNP) may be seen in PASC (310); SLE (371); MIS-C (367); MIS-A (413)

## 4 The impact of SARS-CoV-2 variants

The impact of variants throughout the pandemic is a subject of ongoing interest, particularly concerning ongoing vaccine development (373). Mutations in the S protein are likely to be the most important in determining viral tropism, infectivity, and mortality (374). However, mutations in other proteins, such as R203K and G204R in N, may also impact viral fitness (375). A 2022 study compared the impact of Delta and Omicron SARS-CoV-2 variants in a cohort of 65 patients admitted to an intensive care unit. A higher mortality rate was seen with Omicron (52.9% vs. 41.9% with Delta), although the study acknowledges a higher rate of comorbidities among Omicron patients (376). Similar PaO2/FiO2 ratios (partial pressure of oxygen in arteries to the fraction of inspired oxygen) were seen [Omicron: 156.57, SD 65.98 vs. Delta: 157.31, SD 84.56 (p=0.971)], suggesting a role for extrapulmonary pathophysiology. Organ-specific sequelae may also relate to SARS-CoV-2 variants: Cardiovascular mortality was higher during the pandemic period, ranging from March to June 2020 (likely the D614G variant) and during the Delta wave (June to December 2021) (326). Rates of AKI were higher among Omicron patients than Delta (13 (38.24%) vs. 3 (9.7%), OR 5.78; CI 1.46–22.9, p=0.0172) (376), which may be due to a higher rate of comorbidities among Omicron patients admitted to the ICU rather than being reflective of virus-specific effects.

One meta-analysis examined the impact of variants on post-COVID symptomatology by pooled estimates of several studies, reporting CT abnormalities (60.5%; 95% CI: 40.4-80.6%) and sleep difficulty (24.5%; 95%: 17.5-31.5%) to be the most common PASC symptom following infection with wild-type SARS-CoV-2, fatigue most common among survivors of the Alpha variant, and myalgia among survivors of the Omicron variant (11.7% (95% CI: 8.3-15.1%) in Omicron compared to 9.4% (95% CI: 6.3-12.5%) in wild-type) (377). In that same study, the Alpha and Gamma variants had higher rates of dyspnea (34.2% (8.3-60.1%) and 43.0% (35.3, 50.8%), respectively). The Alpha variant was found to have a higher rate of patients with greater than one general symptom of PASC and fatigue (377). As noted by the authors and as suggested by recent meta-analyses, vaccination is likely to have attenuated the risk of PASC among survivors of COVID-19 (323, 378, 379). As pointed out by the authors of a recent study on pooled survivors of COVID-19 across SARS-CoV-2 variants (323), however, it is difficult to parse out the interaction of vaccines with individual variants, which may be the subject of future studies.

Mechanisms underlying responses to variants of concern may vary. Studies in zebrafish models showed that the wild-type (WT)/Wuhan SARS-CoV-2 variant activated emergency myelopoiesis and recruited neutrophils and macrophages through inflammasome production (380). Deficiency of ACE2 led to exacerbation of inflammation, which was reversed with Ang 1-7 injection, suggesting a role for this pathway in WT-SARS-CoV-2 (380). S1 from the Gamma (P.1) variant (S1γ) and from the Beta variant (S1β from B.1.351) produced higher levels of inflammation, and S1δ (Delta variant; B.1.617.2) produced lower levels of inflammation as measured by macrophage and neutrophil recruitment as well as NF-κB activity in this study (380). A study published in November 2021 showed evidence of microvascular dysfunction among patients infected with SARS-CoV-2 (exact variants unknown) (381), which was not seen in a follow-up study among patients infected with Omicron (382). A complete discussion regarding variant-specific mechanisms of virulence is outside of the scope of this review and will likely be the subject of ongoing study [see, e.g. (375)].

## 5 Conclusions

In conclusion, the immune response to novel SARS-CoV-2 involves a complex milieu of cytokines, macrophages, lymphocytes, and other immune cells skewed towards a pathologically hyperactivated response. Organized but immunostimulatory pyroptosis and inflammasome production may be at play in an environment that lacks crucial early antiviral interferon production, which, along with the development of immune-mediated microthrombi, may be associated with the severity of disease in COVID-19. In the kidney, these mechanisms, as well as acute tubular injury, early myofibroblast activation, and collapsing glomerulopathy in select populations, are likely to account for COVID-19-related AKI and CKD development.

Among the limitations of this review is a thorough discussion regarding variant-specific mechanisms of immune responses, particularly as subsequent waves of the pandemic met longitudinal populations of varying degrees of immunity mediated through vaccination and natural infection. Typical immune responses to SARS-CoV-2 continue to change as the novelty of the virus changes.

It is still unclear whether the acute and long-term sequelae and associated pathophysiology following SARS-CoV-2 infection are unique to the virus or whether this reflects the unprecedented focus of research brought about by the pandemic. In any case, the pace of research in the COVID-19 era has led to a greater understanding of the components of a regulated immune response to SARS-CoV-2 and perhaps to viral infections more generally.
